# Eighteen New Aeruginosamide Variants Produced by the Baltic Cyanobacterium *Limnoraphis* CCNP1324

**DOI:** 10.3390/md18090446

**Published:** 2020-08-27

**Authors:** Marta Cegłowska, Karolia Szubert, Ewa Wieczerzak, Alicja Kosakowska, Hanna Mazur-Marzec

**Affiliations:** 1 Institute of Oceanology, Polish Academy of Sciences, Powstańców Warszawy 55, PL-81712 Sopot, Poland; mceglowska@iopan.pl (M.C.); akosak@iopan.gda.pl (A.K.); 2 Division of Marine Biotechnology, Faculty of Oceanography and Geography, University of Gdańsk, Marszałka J. Piłsudskiego 46, PL-81378 Gdynia, Poland; karolina.szubert@phdstud.ug.edu.pl; 3 Department of Biomedical Chemistry, Faculty of Chemistry, University of Gdańsk, Wita Stwosza 63, PL-80308 Gdańsk, Poland; ewa.wieczerzak@ug.edu.pl

**Keywords:** cyanobacteria, aeruginosamides, *Limnoraphis*, cytotoxicity

## Abstract

Cyanobactins are a large family of ribosomally synthesized and post-translationally modified cyanopeptides (RiPPs). Thus far, over a hundred cyanobactins have been detected in different free-living and symbiotic cyanobacteria. The majority of these peptides have a cyclic structure. The occurrence of linear cyanobactins, aeruginosamides and virenamide, has been reported sporadically and in few cyanobacterial taxa. In the current work, the production of cyanobactins by *Limnoraphis* sp. CCNP1324, isolated from the brackish water Baltic Sea, has been studied for the first time. In the strain, eighteen new aeruginosamide (AEG) variants have been detected. These compounds are characterized by the presence of prenyl and thiazole groups. A common element of AEGs produced by *Limnoraphis* sp. CCNP1324 is the sequence of the three C-terminal residues containing proline, pyrrolidine and methyl ester of thiazolidyne-4-carboxylic acid (Pro-Pyr-TzlCOOMe) or thiazolidyne-4-carboxylic acid (Pro-Pyr-TzlCOOH). The aeruginosamides with methylhomotyrosine (MeHTyr^1^) and with the unidentified *N*-terminal amino acids showed strong cytotoxic activity against human breast cancer cells (T47D).

## 1. Introduction

Nonribosomal and ribosomal cyanobacterial peptides, with their structural diversity and modified amino acid moieties, constitute one of the most interesting and biotechnologically promising groups of marine natural products [1,2,3,4,5]. Ribosomally synthesized and post-translationally modified (RiPPs) cyanobactins constitute a large family of compounds containing from three to twenty amino acids [6,7,8,9]. The biosynthesis of these metabolites starts with the encoding of a precursor peptide that undergoes multiple cleavages leading to a release of a core peptide that is subjected to further enzymatic modifications. The structure of cyanobactins is characterized by the presence of heterocyclized amino acids, mainly cysteine (cyclized to thiazole or oxidized thiazoline), threonine and serine (cyclized to oxazole or oxazoline) [6,7,10]. Cyanobactins can also contain prenyl or, more rarely, geranyl groups. Other modifications include carboxylation of glutamine, hydroxylation of proline, valine or lysine, bromination of tryptophan, acetylation of tyrosine, epimerization or formation of disulfate bridge [7,10,11].

Some cyanobactins, such as comoramides, keenamide A, patellamides and vineramides, exhibit cytotoxic activity against several cancer cell lines [12,13,14,15,16,17]. Venturamides, another class of the peptides, had strong in vitro antimalarial activity against *Plasmodium falciparum* [18]. Cyanobactins have been also described as allelopathic agents. Nostocyclamide from *Nostoc* 31 inhibited the growth of cyanobacterial strains representing other genera *Anabaena*, *Synechococcus* and *Synechocystis*, diatom *Navicula minima* and chlorophyceae *Nannochloris coccoides* [19,20].

The first cyanobactins, ulicyclamide and ulithiacyclamide with cytotoxic activity, were isolated from a tunicate *Lissoclinum patella* from Palau, Western Caroline Islands [21]. It was later established that some cyanobactins were in fact produced by the ascidians symbiont, *Prochloron* spp. [22,23]. Thus far, over a hundred cyanobactins have been detected in different free-living and symbiotic cyanobacteria. Amongst others, these compounds have been found and chemically characterized in *Anabaena* (anacyclamides) [24], *Arthrospira* (arthrospiramides) [25], *Lyngbya* (aesturamides) [26], *Microcystis* (aerucyclamides, aeruginosamides, kawaguchipeptins, microcyclamide, microphycin) [15,27,28,29,30], *Scytonema* (scytodecamide) [31] and *Sphaerospermopsis* (sphaerocyclamides) [32]. Cyanobactin gene clusters were found in up to 30% of cyanobacteria representing *Prochloron*, *Anabaena*, *Microcystis*, *Arthrospira* and other genera [6,7,8,24,33,34].

Initially, cyanobactins were described as cyclic peptides. Lawton et al. [28] reported the production of a linear aeruginosamide by *M. aeruginosa* from bloom sample collected in Rutland Water reservoir (Scotland). This peptide contained the diisoprenylamine and the carboxylated thiazole moieties and was later called aeruginosamide A (AEG-A) [34]. Further studies revealed the presence of modified linear cyanobactins: aeruginosamides B and C in *Microcystis aeruginosa* PCC 9432 and a virenamide A in *Oscillatoria nigro-viridis* PCC 7112 [34].

In the current study, the potential of *Limnoraphis* to produce cyanobactins has been explored for the first time. The non-targeted liquid chromatography-tandem mass spectrometry (LC-MS/MS) analysis of extract and fractions from *Limnoraphis* sp. CCNP1324, isolated from the brackish water Baltic Sea, led to the detection of eighteen new aeruginosamide variants. In cell viability assays some of the aeruginosamides produced by *Limnoraphis* sp. CCNP1324 showed cytotoxic activity against human breast cancer cells (T47D).

## 2. Results and Discussion

The existing knowledge about the structural diversity of aeruginosamides and aeruginosamide-producing cyanobacteria is limited. To date, only three aeruginosamides have been detected [28,34] (Table 1), and no reports on cyanobactins or genes involved in their biosynthesis in cyanobacteria of *Limnoraphis* genus have been published. In our work, the production and structural diversity of cyanobactins produced by *Limnoraphis* sp. CCNP1324 from the Baltic Sea were studied. As a result, eighteen new structural analogues of the linear aeruginosamides were characterized.

Of the eighteen AEGs produced by *Limnoraphis* sp. CCNP1324, the cell-bound content of AEG707, estimated on the basis of chromatographic peak area, was the highest. Ten peptides were produced in trace amounts and were only detected when a larger portion of cyanobacterial biomass was used for the extraction (Table 1). The structure elucidation of AEGs was based on the mass fragmentation spectra with characteristic immonium ions (e.g., at *m/z* 70 (proline Pro), 86 (isoleucine Ile/leucine Leu), 120 (phenylalanine Phe), 134 (homophenylalanine Hph/*N*-methyl-phenylalanine *N*-MePhe), 136 (tyrosine Tyr), 164 (*N*-methyl-homotyrosine *N*-MeHTyr)) and a series of other fragment ions. In addition, the collected product ion spectra were compared with the previously published spectra of AEG-A [28], AEG-B and AEG-C [34].

**Figure 1:**
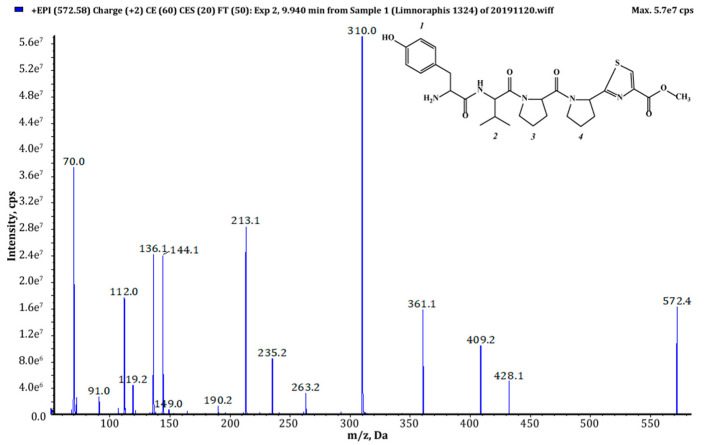
Chemical structure and enhanced product ion mass spectrum of aeruginosamide AEG571 Tyr+Val+Pro+Pyr+TzlCOOMe identified based on the following fragment ions: 572 [M+H], 428 [M+H–TzlCOOMe], 409 [Val+Pro+Pyr+TzlCOOMe], 361 [M+H–Pyr+TzlCOOMe], 310 [Pro+Pyr+TzlCOOMe+H], 263 [Tyr+Val+H], 235 [Tyr+Val+H–CO], 213 [Pyr+TzlCOOMe+H], 144 [TzlCOOMe], 136 Tyr immonium ion, 112 TzlCO, 70 Pro immonium ion.

**Figure 2:**
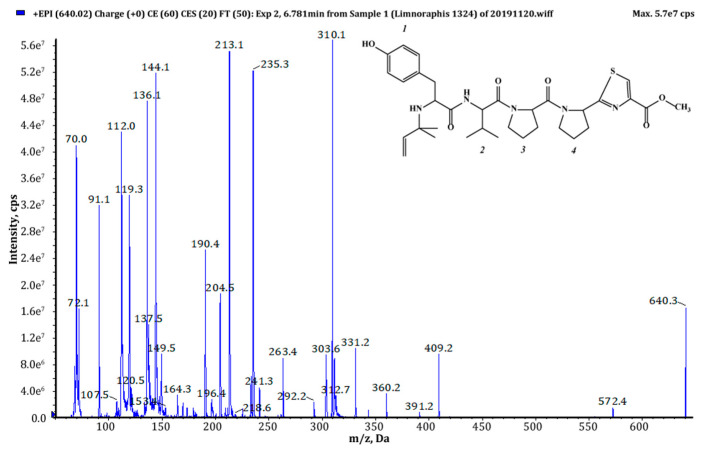
Chemical structure and enhanced product ion mass spectrum of aeruginosamide AEG639 Pre+Tyr+Val+Pro+Pyr+TzlCOOMe identified based on the following fragment ions: 640 [M+H], 572 [M+H–Pre], 409 [Val+Pro+Pyr+TzlCOOMe], 391 [Val+Pro+Pyr+TzlCOOMe–H_2_O], 360 [Tyr+Val+Pro+H], 331 [Pre+Tyr+Val+H], 310 [Pro+Pyr+TzlCOOMe+H], 303 [Pre+Tyr+Val+H–CO], 263 [Tyr+Val+H], 235 [Tyr+Val+H–CO], 213 [Pyr+TzlCOOMe+H], 204 [Pre+Tyr+H–CO], 144 [TzlCOOMe], 136 Tyr immonium ion, 112 TzlCO, 72 Val immonium ion, 70 Pro immonium ion.

**Figure 3:**
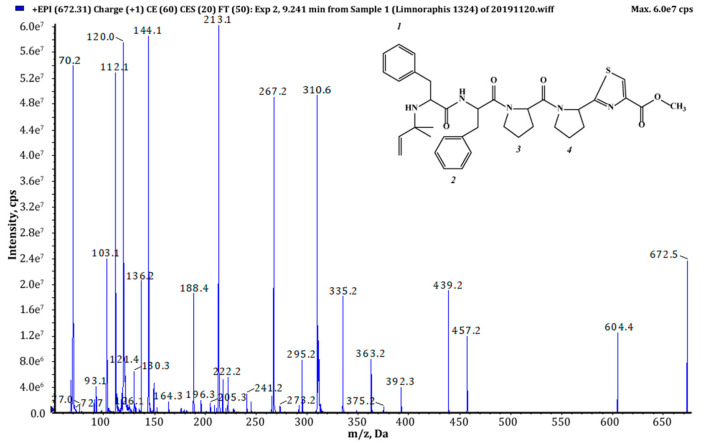
Chemical structure and enhanced product ion mass spectrum of aeruginosamide AEG671 Pre+Phe+Phe+Pro+Pyr+TzlCOOMe identified based on the following fragment ions: 672 [M+H], 604 [M+H–Pre], 457 [M+H–(Pre+Phe)], 439 [M+H–(Pre+Phe)–H_2_O], 392 [Phe+Phe+Pro+H], 363 [Pre+Phe+Phe+H], 335 [Pre+Phe+Phe+H–CO], 310 [Pro+Pyr+TzlCOOMe+H], 295 [Phe+Phe+H], 267 [Phe+Phe+H–CO], 213 [Pyr+TzlCOOMe+H], 188 [Pre+Phe+H–CO], 144 [TzlOMe], 136 Tyr immonium ion, 120 Phe immonium ion; 112 TzlCO, 70 Pro immonium ion.

**Figure 4:**
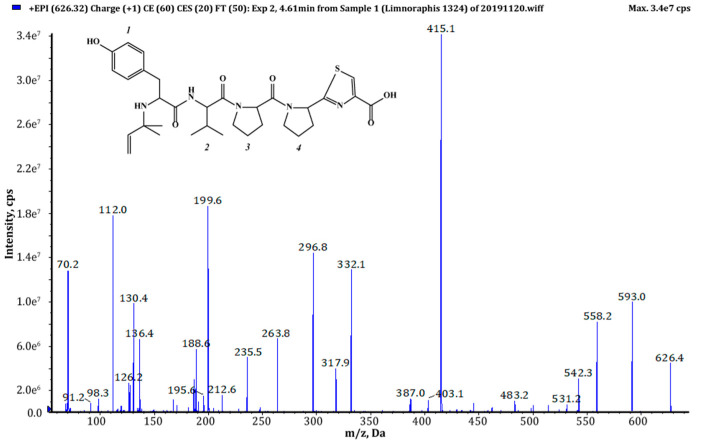
Chemical structure and enhanced product ion mass spectrum of aeruginosamide AEG625 Pre+Tyr+Val+Pro+Pyr+TzlCOOH identified based on the following fragment ions: 626 [M+H], 558 [M+H–Pre], 296 [Pro+Pyr+TzlCOOH+H], 263 [Tyr+Val+H], 332 [Tyr+Val+Pro+H–CO], 235 [Tyr+Val+H–CO], 199 [Pyr+TzlCOOH+H], 130 [TzlCOOH], 136 Tyr immonum ion, 112 TzlCO, 70 Pro immonium ion.

**Figure 5:**
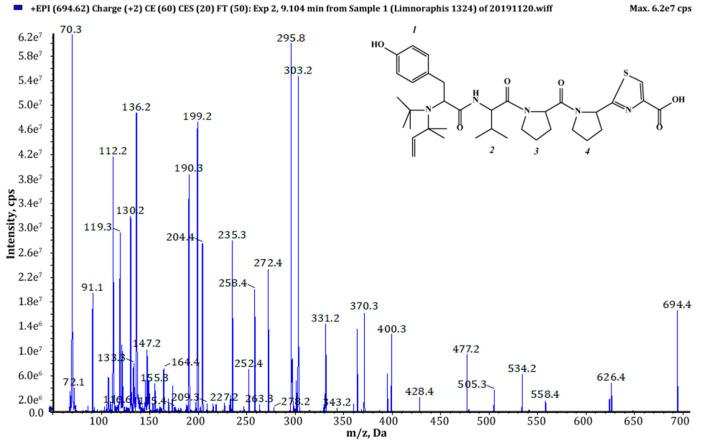
Chemical structure and enhanced product ion mass spectrum of aeruginosamide AEG693 (Pre)_2_+Tyr+Val+Pro+Pyr+TzlCOOH identified based on the following fragment ions: 694 [M+H], 626 [M+H–Pre], 558 [M+H–(Pre)_2_], 477 [(Pre)_2_+Tyr+Val+Pro+H–H_2_O], 428 [Pre+Tyr+Val+Pro+H], 400 [Pre+Tyr+Val+Pro+H–CO], 370 [(Pre)_2_+Tyr+Val+H–CO], 331 [Pre+Tyr+Val+H], 303 [Pre+Tyr+Val+H–CO], 295 [Pro+Pyr+TzlCOOH+H], 272 [(Pre)_2_+Tyr+H–CO], 235 [Tyr+Val+H–CO], 204 [Pre+Tyr+H–CO], 199 [Pyr+TzlCOOH+H], 130 [TzlOH], 136 Tyr immonium ion, 112 TzlCO, 72 Val immonium ion, 70 Pro immonium ion.

Thiazole (Tzl) group, a characteristic element of numerous cyanobactins [6,7,25,35] was present in all AEGs produced by *Limnoraphis* sp. CCNP1324. In the fragmentation spectra, TzlCO gave a peak at *m/z* 112, while the ion at *m/z* 144 was indicative of methyl ester of thiazolidyne-4-carboxylic acid (TzlCOOMe) (Figure 1, Figure 2 and Figure 3, Figures S1, S2, S5–S11 and S13). In the spectra of four AEGs, the ion at *m/z* 112 was present but instead of the *m/z* 144 ion, the ion at *m/z* 130 occurred, suggesting a modification in the ester group of TzlCOOMe. In the spectra of these peptides, instead of ions at *m/z* 213 (Pyr+TzlCOOMe) and *m/z* 310 (Pro+Pyr+TzlCOOMe), there were peaks at 14 units lower values, i.e., *m/z* 199 and 296. Pyr stands for pyrrolidine ring which constitutes a part of the proline structure. The 14-unit shift in the *m/z* value of the ions, compared to TzlCOOMe-containing peptides, and the ion at *m/z* 112 indicated the presence of thiazolidyne-4-carboxylic acid (TzlCOOH). Such modifications were observed in AEG625 (Figure 4), AEG657 (Figure S3), AEG693 (Figure 5) and AEG735 (Figure S12) (Table 1). The three *C*-terminal residues in aeruginosamides identified in CCNP1324, were found to be conserved. In other AEGs identified thus far the residues adjacent to TzlCOOMe were Val (valine)+Pyr [28], Phe+Pyr or Pro+Val [34] (Table 1).

Tyr^1^ was found to be the most frequent residue at the *N*-terminus and was present in six out of eighteen AEGs identified in this study. In other AEGs produced by *Limnoraphis* sp. CCNP1324, this position was occupied by MeHTyr^1^, Phe^1^ or Hph^1^ (Table 1, Figure 1, Figure 2, Figure 3, Figure 4 and Figure 5 and Figures S1–S13). In the case of six AEGs (*m/z* [M+H] 596, 682a, 682b, 684, 736 and 750) we were not able to fully elucidate the structure and identify the *N*-terminal residue. Based on the fragmentation spectrum it was concluded that the residues gave strong immonium ions at *m/z* 160, 178, 180 and 198 and their residue masses were 187, 205, 207 and 225 respectively. In previously described linear cyanobactins such as virenamide A–C, aeruginosamide B and C, and viridisamide A, Phe^1^ was the most commonly identified *N*-terminal residue [12,34]. In other cyanobactins, position 1 was occupied by Ile [28,36] or Val [37]. The high residue masses of the unidentified amino acids and a frequent occurrence of aromatic amino acids at *N*-terminus of AEGs produced by *Limnoraphis* sp. indicated the presence of modified Tyr or Phe variants in this position. In some RiPPs, such as cyanobactins and microviridins, the presence of acetylated Tyr (AcTyr) was reported [11,38]. Based on the mass fragmentation spectrum, the presence of AcTyr^1^ in AEG681a is also possible (Figure S5). The position 2 in AEGs produced by *Limnoraphis* sp. CCNP1324 was least conserved and occupied by both aliphatic and aromatic amino acids: Val, Ile, Phe and Hph/MePhe (Table 1, Figure 1, Figure 2, Figure 3, Figure 4 and Figure 5 and Figures S1–S13).

*Limnoraphis* sp. CCNP1324 synthesizes aeruginosamides with two, one and no prenyl groups at *N*-terminus (Table 1, Figure 1, Figure 2, Figure 3, Figure 4 and Figure 5 and Figures S1–S13). The presence of prenyl was confirmed by the loss of one or two 68-Da fragments from the pseudomolecular ion of the analyzed peptides. The differences in retention times between AEGs without and with prenyl group (Table 1), indicate that the former ones are not the products of in-source degradation. In other cyanobactins, the number of Pre groups also varied depending on the peptide. Doubly prenylated cyanobactin, virenamide A, was reported from *D. virens* [12], while monoprenylated AEG-B, AEG-C, viridisamide A [34] and virenamide B and C [12] were identified in *M. aeruginosa* PCC9432, *O. nigro viridis* PCC7112 and *D. virens*, respectively. Prenyl groups at both C- and *N*-terminus were found in muscoride A and B from *N. muscorum* IAM M-14, *Nostoc* sp. PCC7906 and *Nostoc* sp. UMCC0398 [36,37].

**Figure 6:**
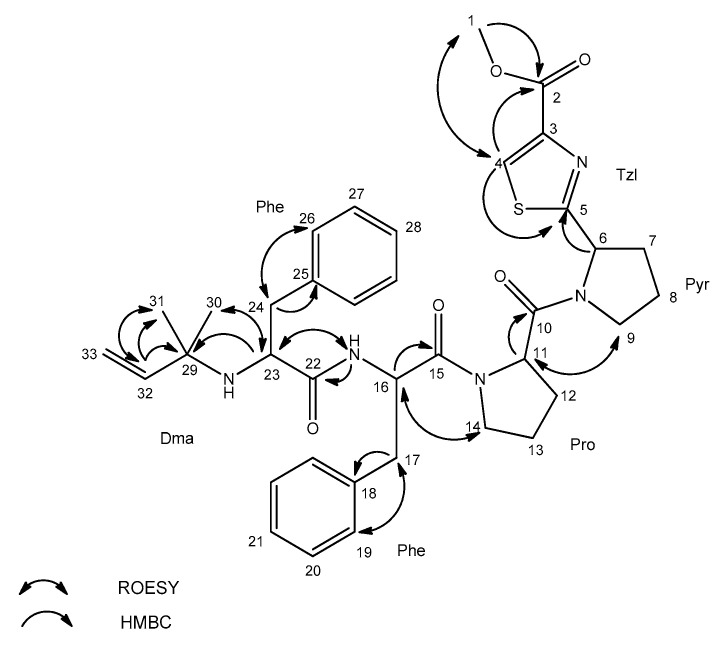
Key Rotation Frame Nuclear Overhauser Effect Spectroscopy ROESY and Heteronuclear Multiple Bond Coherence HMBC correlations for aeruginosamide AEG671.

Due to the chromatographic behaviour of AEG671, which allowed for the isolation of the peptide (1 mg) as a pure compound, the structural analyses with application of Nuclear Magnetic Resonance (NMR) were possible. Unfortunately, under the chromatographic conditions used in the current study, the majority of the detected aeruginosamides were poorly separated. They occurred in the chromatograms as broad peaks or/and co-eluted with other components of *Limnoraphis* extract. The NMR analyses of the isolated AEG671 confirmed the correctness of structure elucidation performed based on the MS/MS fragmentation pattern of pseudomolecular ion. The ^1^H NMR spectrum of the studied compound displayed a typical pattern of a peptide. The Correlation Spectroscopy COSY, Total Correlation Spectroscopy TOCSY and Heteronuclear Multiple Bond Correlation HMBC data (Figures S14–S19) allowed for the identification of the residues in AER671 as Dma (Dma = 1, 1-dimethylallyl), Phe, Phe, Pro, Pyr and TzlCOOMe (Table 2, Figure 6). Proton and carbon chemical shifts unambiguously showed that the prenyl group in the studied compound was in reverse prenyl, 1, 1-dimethylallyl form.

The signals occurring in the aromatic region of the spectrum (δ_H_ 7.1–7.5 ppm) and the TOCSY interaction between 19 (26), 20 (27) and 21 (28) protons were indicative of the presence of two aromatic phenylalanine residues in the molecule. The existence of proline residue and pyrrolidine ring was confirmed by their characteristic spin systems in the TOCSY spectrum. HMBC correlation of proton 6 (δ_H_ 5.32 ppm) to thiazole carbon 5 (δ_C_ 173.4 ppm) confirmed the connection of Pyr to Tzl ring. The presence of methyl thiazole-carboxylate was shown by characteristic proton (δ_H_ 3.81 ppm) and carbon (δ_C_ 51.1 ppm) chemical shifts and HMBC correlation of methyl protons 1 (δ_H_ 3.81 ppm) to carbon 2 (δ_C_ 160.3 ppm), and by HMBC and Heteronuclear Single Quantum Correlation HSQC of proton 4 (δ_H_ 8.43 ppm) to carbons 2 (δ_C_ 160.3 ppm), 5 (δ_C_ 173.4 ppm), and 4 (δ_C_ 128.0 ppm).

Apart from reversed prenyl as present in AEG671, cyanobactins can also contain a forward prenylated *N*-terminus (e.g., AEG-A [28] and virenamide A [12]), as well as, a forward C-, and reverse prenylated *N*-terminus (muscoride A [36]) or forward prenylated both C- and *N*-termini (muscoride B [37]).

Protein prenylation is an important posttranslational modification which increases the lipophilicity and affinity of compounds for biological membranes [39,40,41]. Prenylation also increases the biological activity of natural products [42,43]. The cytotoxic activities of prenylated licoflavone C and isobavachinas from plants, as well as their non-prenylated analogues (apigenin, liquiritigenin), were examined against glioma (C6) and rat hepatoma (H4IIE) cells. The prenylated compounds showed pronounced cytotoxicity against both types of cells while their non-prenylated analogues were weakly active [42].

The activity of cyanobactins and cyanobactin-like peptides has been tested against bladder carcinoma (T24), colon adenocarcinoma (HT29), lung carcinoma (A549) and murine leukemia (P388) cell lines, proving the pharmacological potential of these compounds [12,13,14,15,16,17]. Cyanobactins also showed multidrug-resistance reversing activity [44].

The existing knowledge about the activity of aeruginosamides is scarce. To date, only mild cytotoxic effects of aeruginosamide A against human ovarian tumor (A2780) and human leukemia (K562) cells have been reported [28]. In our work, the cytotoxic activity of three chromatographically separated samples labelled as A, B and C, was tested against T47D cancer cells. The sample marked as A contained AEG671, sample B contained partially separated AEG681a and, in sample C, a mixture of AEG681a and AEG667 was present. After 24-h exposure, sample B containing partially separated AEG681a with unknown residue in position 1 (residue mass 205) reduced the relative cell viability to 4.2% ± 0.5% at 200 µg mL^−1^. Sample C, containing a mixture of AEG681a and AEG667 (with MeHTyr^1^), reduced the relative cell viability to 21% ± 1.2% at 200 µg mL^−1^. These effects were dose dependent. No activity was observed for Phe^1^ containing AEG671 present in sample A. Unfortunately, the cytotoxic peptides with the unidentified residues are produced by *Limnoraphis* sp. CCNP1324 in minute amounts (Table 1), which seriously restricts the ability to perform more detailed structural analyses with the application of NMR technique.

The vast structural diversity of AEGs, as well as the cytotoxic activity of some of the variants, create an opportunity for more detailed studies on the structure-activity relationship. Several cyanobacterial peptides are already in clinical or pre-clinical trials as potent anti-cancer agents [45]. The most successful was the development of Auristatine (brentuximab vedotin), a synthetic analogue of dolastatin 10 isolated from *Dolabella auricularia*, but actually produced by the cyanobacterium *Symploca* sp. [46]. This microtubule-impacting agent was approved by the Food and Drug Administration (FDA), and is globally used in the treatment of Hodgkin’s lymphoma [47].

## 3. Materials and Methods

*Limnoraphis* sp. CCNP1324 was isolated from the Puck Bay in the Southern Baltic Sea (54.45 N, 18.30 E) by Dr. Justyna Kobos in 2012. The strain was obtained from the Culture Collection of Northern Poland (CCNP) at the University of Gdańsk and grown in F/2 medium (7 PSU), at 22 °C ± 0.5, with constant illumination (10 µM photons m^−2^ s^−1^) provided by standard cool white fluorescent lamps.

### 3.1. Extraction and Isolation

Freeze-dried *Limnoraphis* CCNP1324 cells were homogenized using mortar and pestle. The ground cyanobacterial biomass (10 mg) was extracted with 75% methanol in MilliQ water (1 mL) by vortexing (5 min). The sample was then centrifuged (10,000× *g*; 15 min; 4 °C) and the content of aeruginosamides in the obtained supernatant was analyzed using LC-MS/MS.

For fractionation and isolation of aeruginosamides, the homogenized biomass (20 g) was extracted twice with 75% methanol in MilliQ water (2 × 500 mL) by vortexing (20 min). After centrifugation (4000× *g*; 15 min; 4 °C), the supernatants were combined and diluted with MilliQ water, so that the final concentration of MeOH in the extract was <10%. For flash and preparative chromatography a Shimadzu HPLC system model LC-20AP (Shimadzu, Canby, OR, USA) equipped with isocratic and binary pumps, a fraction collector and photodiode array detector (PDA) was used. PDA operated in a range from 190 nm to 500 nm and, during all chromatographic runs, the absorbance at 210 nm and 280 nm was recorded.

To perform flash chromatography, the aqueous methanol extract (MeOH < 10%) was loaded onto a preconditioned 120 g SNAP KP-C_18_-HS cartridge (Biotage Uppsala, Sweden) using an isocratic pump, at a flow rate of 15 mL min^−1^. Components of the extract were separated with a mixture of a mobile phase composed of MilliQ water (A_1_) and 100% MeOH (B_1_). The gradient started at 10% B_1_ and went to 30% B_1_ within 20 min. After 90 min, the content of B_1_ increased to 70% and was kept at that level for 10 min before increasing to 100% B_1_ within the next 30 min. The flow rate of the eluent was 20 mL m^−1^ and 50 mL fractions were collected.

AEGs-containing flash fractions, eluted with 86–93% B_1_ (Prep1), and 58–68% B_1_ (Prep2), were combined and concentrated in a centrifugal vacuum concentrator (MiVac, SP Scientific, Ipswich, UK). Dried samples (Prep1 and Prep2) were first solubilized using 0.6 mL of 60% MeOH, followed by 0.6 mL of 20% MeOH. After centrifugation, the supernatants were loaded onto a preparative column using the Rheodyne injector. The sample components were separated on a Jupiter Proteo C_12_ column (250 × 21.2 mm; 4 μm; 90 Å) (Phenomenex, Aschaffenburg, Germany) with a mobile phase composed of 5% acetonitrile in MilliQ water (A_2_) and acetonitrile (B_2_), both with the addition of 0.1% formic acid. During the separation process the flow rate was 20 mL m^−1^ and 4 mL fractions were collected.

In the case of fractions 86–93% (Prep1), the gradient started at 20% B_2_, then went to 30% B_2_ in 25 min, after 10 min B_2_ reached 90%. After another 2 min, B_2_ increased to 100% and was kept at that level for 13 min. Fractions eluted with 25–27% B_2_ (vials 75–103) and containing an isolated single peak were pulled, vacuum concentrated and marked as sample A (1 mg). Fractions eluted with 23–25% B_2_ (vials 40–74), which also corresponded to a single peak in HPLC-PDA chromatogram, were pulled, evaporated to dryness and marked as sample B (0.9 mg).

The preparative separation of flash fractions 58–68% B_1_ (Prep2) started at 15% B_2_ and went to 30% B_2_ in 20 min, after 10 min B_2_ reached 90%. After another 2 min, B_2_ increased to 100% and was kept at that level for 8 min. Fractions eluted with 24–27% B_2_ (with AEGs) were prepared as described above and subjected to further separation. In the subsequent run, the gradient started at 5% B_2_ and went to 40% B_2_ in 20 min, after 5 min B_2_ reached 100% and was kept at that level for 5 min. Fractions eluted with 27–37% B_2_, containing a single peak were pulled evaporated to dryness and marked as sample C (1.2 mg). The samples A, B and C were subjected to LC-MS/MS analyses and cytotoxicity assays. For sample A, the NMR analyses were additionally performed.

### 3.2. LC-MS/MS Analysis

The contents of cyanobacterial extracts, fractions and isolated compounds, were analyzed with the application of an Agilent 1200 (Agilent Technologies, Waldboronn, Germany) HPLC system coupled with a hybrid triple quadrupole/linear ion trap mass spectrometer (QTRAP5500, Applied Biosystems, Sciex, Concorde, ON, Canada). For peptide separation a Zorbax Eclipse XDB-C_18_ column (4.6 × 150 mm; 5 μm) (Agilent Technologies, Santa Clara, CA, USA) was used. The mobile phase was composed of a mixture of 5% acetonitrile in MilliQ water (A_2_) and acetonitrile (B_2_), both with the addition of 0.1% formic acid. A gradient elution at 0.6 mL min^−1^ was applied. The system operated in positive mode with a turbo ion spray (550 °C; 5.5 kV). The non-targeted information-dependent acquisition (IDA) mode was applied to screen the content of the samples. Fragmentation spectra of ions within the *m/z* range 400–1000, and signal intensity above 500,000 cps were collected, at a collision energy of 60 ± 20 eV. The structures of aeruginosamides were additionally characterized using targeted enhanced product ion (EPI) mode.

### 3.3. NMR Analysis

The 1D ^1^H NMR and 2D homo- and heteronuclear NMR (COSY, TOCSY, ROESY, HSQC, and HMBC) were acquired with the application of a Varian Unity Inova 500 spectrometer (500 MHz). Spectra were recorded in dimethyl sulfoxide-d_6_ (DMSO-d_6_). NMR data were processed and analyzed by TopSpin (Bruker, Billerica, MA, USA) and SPARKY software (3.114, Goddard and Kneller, freeware (https://www.cgl.ucsf.edu/home/sparky).

### 3.4. Cytotoxicity Assays

The cytotoxic activity of the isolated and identified AEG671 as well as the activity of two other samples containing AEGs as the main components was tested. For the purpose the 3-(4,5-dimethylthiazol-2-yl)-2,5-diphenyltetrazolium-bromide (MTT) assays with the application of a human breast adenocarcinoma cell line T47D (Merck KGaA, Darmstadt, Germany) were performed as described by Felczykowska et al. [48] and Szubert et al. [49]. T47D cells were plated at 1 × 10^4^ cells per well of 96-well plate containing RPMI1640 (Carl Roth GmbH) medium supplemented with 10% fetal bovine serum (Merck KGaA) and penicillin-streptomycin solution (50 u and 0.05 mg per 1 mL of medium respectively; Merck KGaA) (24 h at 37 °C, 5% CO_2_). The cytotoxic effects of tested samples dissolved in 1% DMSO, at final concentrations 25, 50, 100 and 200 µg ml^−1^ (in culture medium) were examined after 24 h incubation (37 °C, 5% CO_2_) using a microplate reader (Spectramax i3, Molecular Devices, LLC. San Jose, CA, USA). Cell viability was calculated as the ratio of the mean absorbance value, for the six replicates containing the samples, to the mean absorbance of the six replicates of the corresponding solvent control, and expressed as a percentage. The results were considered as significant when cell viability decreased below 50%.

## 4. Conclusions

In this work, *Limnoraphis* sp. CCNP1324 was revealed to be a new producer of aeruginosamides. Some of the peptides were cytotoxic against a breast cancer cell line. The cytotoxic activity of these compounds is probably determined by the unknown amino acid residues in *N*-terminal position. Unfortunately, the data collected with MS/MS were insufficient to resolve their structures. LC-MS/MS analyses of samples are key elements of bioassay-guided fractionation and structure characterization of bioactive metabolites. Due to high sensitivity and selectivity, trace amounts of the compounds in complex matrices can be detected. However, like any technique, it has also some limitations. The unequivocal elucidation of peptide structure with unknown modifications is impossible or bears a high risk of error. Therefore, in our future work, the chromatographic conditions have to be further optimized, to isolate the bioactive peptides in sufficient amounts for structural analysis by NMR.

## Figures and Tables

**Table 1:** Postulated structures of aeruginosamides (AEGs) described thus far, and identified in *Limnoraphis* sp. CCNP1324.

Aeruginosamide AEG	*m/z*	Retention Time [min]	Relative Peak Area of Extracted Ion	AEG Chemical Structure	Reference
AEG-A	561	-	-	(Pre)_2_+Ile+Val+Pyr+TzlCOOMe	[28]
571 ^(1),(2)^	572	9.94	1.39 × 10^9^	Tyr+Val+Pro+Pyr+TzlCOOMe	This study
AEG-B	575	-	-	Pre+Phe+Phe+Pyr+TzlCOOMe	[34]
595 ^(2)^	596	5.12	T	187+Val+Pro+Pyr+TzlCOOMe	This study
603 ^(2)^	604	9.74	T	Phe+Phe+Pro+Pyr+TzlCOOMe	This study
625 ^(1),(2)^	626	4.61	6.82 × 10^8^	Pre+Tyr+Val+Pro+Pyr+TzlCOOH	This study
639 ^(1),(2)^	640	6.78	1.23 × 10^9^	Pre+Tyr+Val+Pro+Pyr+TzlCOOMe	This study
657 ^(1),(2)^	658	2.27	5.8 × 10^8^	Pre+Phe+Phe+Pro+Pyr+TzlCOOH	This study
667 ^(2)^	668	8.22	T	Pre+MeHTyr+Val+Pro+Pyr+TzlCOOMe	This study
671 ^(1),(2)^	672	9.24	4.45 × 10^8^	Pre+Phe+Phe+Pro+Pyr+TzlCOOMe	This study
AEG-C	674	-	-	Pre+Phe+Phe+Pro+Val+TzlCOOMe	[34]
681a ^(2)^	682a	7.16	T	Pre+205+Val+Pro+Pyr+TzlCOOMe	This study
681b ^(2)^	682b	11.78	T	225+Phe+Pro+Pyr+TzlCOOMe	This study
683 ^(2)^	684	12.34	T	Pre+207+Val+Pro+Pyr+TzlCOOMe	This study
685 ^(2)^	686	12.62	T	Pre+Phe+Hph/MePhe+Pro+Pyr+TzlCOOMe	This study
693 ^(1),(2)^	694	9.10	2.85 × 10^9^	(Pre)_2_+Tyr+Val+Pro+Pyr+TzlCOOH	This study
705 ^(2)^	706	8.73	T	(Pre)_2_+Hph/MePhe+Val+Pro+Pyr+TzlCOOMe	This study
707 ^(1),(2)^	708	9.76	8.69 × 10^9^	(Pre)_2_+Tyr+Val+Pro+Pyr+TzlCOOMe	This study
721 ^(1),(2)^	722	10.12	1.89 × 10^8^	(Pre)_2_+Tyr+Ile/Leu+Pro+Pyr+TzlCOOMe	This study
735 ^(2)^	736	11.26	T	Pre+225+Phe+Pro+Pyr+TzlCOOH	This study
749 ^(2)^	750	11.50	T	Pre+225+Phe+Pro+Pyr+TzlCOOMe	This study

^(1)^ Detected in the 10 mg extract. ^(2)^ Detected in the 20 g extract and flash fractions from CCNP1324.T traces of AEGs detected in 10 mg extract. Hph: homophenylalanine; MePhe: *N*-methy-phenylalanine; Ile/Leu: isoleucine/leucine; Phe: phenylalanine, Pre: prenyl group; Pro: proline; Pyr: pyrrolidine; Tyr: tyrosine; MeHTyr: *N*-methyl-homotyrosine; TzlCOOH: thiazolidyne-4-carboxylic acid; TzlCOOMe: methyl ester of thiazolidyne-4-carboxylic acid; Val: valine; 187, 205, 225: unknown residues.

**Table 2:** Nuclear Magnetic Resonance NMR Spectroscopic Data (500 MHz, dimethyl sulfoxide-d_6_ DMSO-d_6_) for aeruginosamide AEG371 (Dma-Phe-Phe-Pro-Pyr-Tzl-COOMe).

Residue	Position	ä_C_, Type	ä_H_ (*J* in Hz)	ROESY ^a^	HMBC ^b^
**Tzl-COOMe**	1	51.1, CH_3_	3.81, s	4	2
	2	160.3, C			
	3	144.5, C			
	4	128.0, CH	8.43, s	1	2, 5
	5	173.4, C			
**Pyr**	6	57.5, CH	5.32, dd (8.2, 2.4)		5
	7	30.5, CH_2_	2.05, m		
			2.27, m		
	8	23.3, CH_2_	1.87, m		
			1.96, m		
	9	45.9, CH_2_	3.70, m	11	
**Pro**	10	169.7, C			
	11	56.7, CH	4.68, brs	9	10
	12	27.1, CH_2_	1.81, m		
			2.20, m		
	13	23.7, CH_2_	2.04, m		
	14	46.0, CH_2_	3.50, m	16	
			3.66, m		
**Phe**	15	168.2, C			
	16	50.0, CH	4.77, brs	14	15, 18
	17	36.1, CH_2_	2.76, dd (14.1, 8.8)	19	15, 16, 18
			2.97, dd (14.1, 4.3)		
	18	136.3, C			
	19	128.6, CH	7.11, d (7.1)	17	
	20	127.1, CH	7.23, m		
	21	125.3, CH	7.18, m		
	NH(1)		8.18, d (8.9)	23	22
**Phe**	22	173.3, C			
	23	57.1, CH	3.03, dd (9.0, 4.2)	30, NH(1)	29
	24	39.6, CH_2_	2.35, dd (13.3, 9.0)	26	22, 23, 25
			2.67, dd (13.3, 4.1)		
	25	137.4, C			
	26	128.6, CH	7.11, d (7.1)	24	
	27	127.1, CH	7.23, m		
	28	125.4, CH	7.18, m		
	NH(2)				
**Dma ^c^**	29	53.2, C			
	30	24.7, CH_3_	0.79, s	23	
	31	26.6, CH_3_	0.76, s	32	
	32	144.9, CH	5.26, dd (17.5, 10.7)	31	29, 31
	33	110.9, CH_2_	4.72, m		

^a^ ROESY Rotation Frame Nuclear Overhauser Effect Spectroscopy; ^b^ HMBC correlations are given from proton(s) stated to the indicated carbon atom; ^c^ Dma: 1,1-dimethylallyl.

## Supplementary Materials

The following are available online at https://www.mdpi.com/1660-3397/18/9/446/s1, Figures S1–S13. Enhanced product ion mass spectrum of aeruginosamides: AER595, AEG603, AEG657, AER667, AER681a, AER681b, AER683, AER685, AEG705, AEG707, AEG721, AER735, AER749, Figure S14. ^1^H NMR Spectrum of aeruginosamide AEG671 in DMSO-d_6_, Figure S15. COSY Spectrum of aeruginosamide AEG671 in DMSO-d_6_, Figure S16. TOCSY Spectrum of aeruginosamide AEG671 in DMSO-d_6_, Figure S17. ROESY Spectrum of aeruginosamide AEG671 in DMSO-d_6_, Figure S18. HSQC Spectrum of aeruginosamide AEG671 in DMSO-d_6_, Figure S19. HMBC Spectrum of aeruginosamide AEG671 in DMSO-d_6_.

## References

[1] Burja A.M., Banaigs B., Abou-Mansour E., Burgess J.G., Wright P.C. (2001). Marine cyanobacteria—A prolific source of natural products. Tetrahedron.

[2] Chlipala G.E., Mo S., Orjala J. (2011). Chemodiversity in freshwater and terrestrial cyanobacteria—A source for drug discovery. Curr. Cancer Drug Targets.

[3] Dittmann E., Gugger M., Sivonen K., Fewer D.P. (2015). Natural product biosynthetic diversity and comparative genomics of the cyanobacteria. Trends Microbiol..

[4] Shah S.A.A., Akhter N., Auckloo B.N., Khan I., Lu Y., Wang K., Wu B., Guo Y. (2017). Structural diversity, biological properties and applications of natural products from cyanobacteria: A review. Mar. Drugs.

[5] Seddek N.H., Fawzy M.A., El-Said W.A., Ahmed M.M.R. (2019). Evaluation of antimicrobial, antioxidant and cytotoxic activities and characterization of bioactive substances from freshwater blue-green algae. Glob. NEST J..

[6] Sivonen K., Leikoski N., Fewer D.P., Jokela J. (2010). Cyanobactins—Ribosomal cyclic peptides produced by cyanobacteria. Appl. Microbiol. Biotechnol..

[7] Martins J., Vasconcelos V. (2015). Cyanobactins from cyanobacteria: Current genetic and chemical state of knowledge. Mar. Drugs.

[8] Donia M.S., Ravel J., Schmidt E.W. (2008). A global assembly line for cyanobactins. Nat. Chem. Biol..

[9] Gu W., Dong S.H., Sarkar S., Nair S.K., Schmidt E.W. (2018). The biochemistry and structural biology of cyanobactin pathways: Enabling combinatorial biosynthesis. Methods Enzymol..

[10] Arnison P.G., Bibb M.J., Bierbaum G., Bowers A.A., Bugni T.S., Bulaj G., Camarero J.A., Campopiano D.J., Challis G.L., Clardy J. (2013). Ribosomally synthesized and post-translationally modified peptide natural products: Overview and recommendations for a universal nomenclature. Nat. Prod. Rep..

[11] McIntosh J.A., Donia M.S., Nair S.K., Schmidt E.W. (2011). Enzymatic basis of ribosomal peptide prenylation in cyanobacteria. J. Am. Chem. Soc..

[12] Carroll A.R., Feng Y., Bowden B.F., Coll J.C. (1996). Studies of Australian ascidians. 5. Virenamides A–C, new cytotoxic linear peptides from the colonial didemnid ascidian *Diplosoma Virens*. J. Org. Chem..

[13] Wesson K.J., Hamann M. (1996). Keenamide A, a bioactive cyclic peptide from the marine mollusc *Pleurobranchus forskalii*. J. Nat. Prod..

[14] Rudi A., Aknin M., Gaydou E.M., Kashman Y. (1998). Four new cytotoxic cyclic hexa- and heptapeptides from the marine ascidian *Didemnum molle*. Tetrahedron.

[15] Ishida K., Nakagawa H., Murakami M. (2000). Microcyclamide, a cytotoxic cyclic hexapeptide from the cyanobacterium *Microcystis aeruginosa*. J. Nat. Prod..

[16] Ireland C.M., Durso A.R., Newman R.A., Hacker M.P. (1982). Antineoplastic cyclic peptides from the marine tunicate *Lissoclinum patella*. J. Org. Chem..

[17] Degnan B.M., Hawkins C.J., Lavin M.F., McCaffrey E.J., Parry D.L., van den Brenk A.L., Watterst D.J. (1989). New cyclic peptides with cytotoxic activity from the ascidian *Lissoclinum patella*. J. Med. Chem..

[18] Linington R.G., Gonzàles J., Ureña L.-D., Romero L.I., Ortega-Barria E., Gerwick W.H. (2007). Venturamides A and B: Antimalarial constituents of the Panamanian marine cyanobacterium *Oscillatoria* sp.. J. Nat. Prod..

[19] Todorova A.K., Jüttner F., Linden A., Plüss T., von Philipsborn W. (1995). Nostocyclamide: A new macrocyclic, thiazole-containing allelochemical from *Nostoc* sp. 31 (cyanobacteria). J. Org. Chem..

[20] Jüttner F., Todorova A.K., Walch N., von Philipsborn W. (2001). Nostocyclamide M: A cyanobacterial cyclic peptide with allelopathic activity from *Nostoc* 31. Phytochemistry.

[21] Ireland C., Scheuer P.J. (1980). Ulicyclamide and ulithiacyclamide, two new small peptides from a marine Tunicate. J. Am. Chem. Soc..

[22] Long P.F., Dunlap W.C., Battershill C.N., Jaspars M. (2005). Shotgun cloning and heterologous expression of the patellamide gene cluster as a strategy to achieving sustained metabolite production. Chem. Biol. Chem..

[23] Schmidt E.W., Nelson J.T., Rasko D.A., Sudek S., Eisen J.A., Haygood M.G., Ravel J. (2005). Patellamide A and C biosynthesis by a microcin-like pathway in *Prochloron didemni*, the cyanobacterial symbiont of *Lissoclinum patella*. Proc. Natl. Acad. Sci. USA.

[24] Leikoski N., Fewer D.P., Jokela J., Wahlsten M., Rouhiainen L., Sivonen K. (2010). Highly diverse cyanobactins in strains of the genus *Anabaena*. Appl. Environ. Microbiol..

[25] Donia M.S., Schmidt E.W. (2011). Linking chemistry and genetics in the growing cyanobactin natural products family. Chem. Biol..

[26] McIntosh J.A., Lin Z., Tianero M.D., Schmidt E.W. (2013). Aestuaramides, a natural library of cyanobactin cyclic peptides resulting from isoprene-derived Claisen rearrangements. ACS Chem. Biol..

[27] Ishida K., Matsuda H., Murakami M., Yamaguchi K. (1997). Kawaguchipeptin B, an antibacterial cyclic undecapeptide from the cyanobacterium *Microcystis aeruginosa*. J. Nat. Prod..

[28] Lawton L.A., Morris L.A., Jaspars M. (1999). A bioactive modified peptide, aeruginosamide, isolated from the cyanobacterium *Microcystis aeruginosa*. J. Org. Chem..

[29] Gesner-Apter S., Carmeli S. (2008). Three novel metabolites from a bloom of the cyanobacterium *Microcystis* sp.. Tetrahedron.

[30] Portmann C., Blom J.F., Kaiser M., Brun R., Jüttner F., Gademann K. (2008). Isolation of aerucyclamides C and D and structure revision of microcyclamide 7806A: Heterocyclic ribosomal peptides from *Microcystis aeruginosa* PCC 7806 and their antiparasite evaluation. J. Nat. Prod..

[31] Crnkovic C.M., Braesel J., Krunic A., Eustáquio A.S., Orjala J. (2020). Scytodecamide from the cultured *Scytonema* sp. UIC 10036 expands the chemical and genetic diversity of cyanobactins. ChemBioChem.

[32] Martins J., Leikoski N., Wahlsten M., Azevedo J., Antunes J., Jokela J., Sivonen K., Vasconcelos V., Fewer D.P., Leão P.N. (2018). Sphaerocyclamide, a prenylated cyanobactin from the cyanobacterium *Sphaerospermopsis* sp. LEGE 00249. Sci. Rep..

[33] Leikoski N., Fewer D.P., Sivonen K. (2009). Widespread occurrence and lateral transfer of the cyanobactin biosynthesis gene cluster in cyanobacteria. Appl. Environ. Microbiol..

[34] Leikoski N., Liu L., Jokela J., Wahlsten M., Gugger M., Calteau A., Permi P., Kerfeld C.A., Sivonen K., Fewer D.P. (2013). Genome mining expands the chemical diversity of the cyanobactin family to include highly modified linear peptides. Chem. Biol..

[35] Donia M.S., Schmidt E.W. (2010). Comprehensive natural products II chemistry and biology. Cyanobactins—Ubiquitous Cyanobacterial Ribosomal Peptide Metabolites.

[36] Nagatsu A., Kajitani H., Sakakibara J. (1995). Muscoride A: A new oxazole peptide alkaloid from freshwater cyanobacterium *Nostoc muscorum*. Tetrahedron Lett..

[37] Mattila A., Andsten R.M., Jumppanen M., Assante M., Jokela J., Wahlsten M., Mikula K.M., Sigindere C., Kwak D.H., Gugger M. (2019). Biosynthesis of the bis-prenylated alkaloids muscoride A and B. ACS Chem. Biol..

[38] Ziemert N., Ishida K., Liaimer A., Hertweck C., Dittmann E. (2008). Ribosomal synthesis of tricyclic depsipeptides in bloom-forming cyanobacteria. Angew. Chem. Int. Ed. Engl..

[39] Botta B., Vitali A., Menendez P., Misiti D., Monache G. (2005). Prenylated flavonoids: Pharmacology and biotechnology. Curr. Med. Chem..

[40] Wang M., Casey P.J. (2016). Protein prenylation: Unique fats make their mark on biology. Nat. Rev. Mol. Cell Biol..

[41] Wong C.P., Awakawa T., Nakashima Y., Mori T., Zhu Q., Liu X., Abe I. (2018). Two distinct substrate binding modes for the normal and reverse prenylation of hapalindoles by the prenyltransferase AmbP3. Angew. Chem. Int. Ed. Engl..

[42] Wätjen W., Weber N., Lou Y.J., Wang Z.Q., Chovolou Y., Kampkötter A., Kahl R., Proksch P. (2007). Prenylation enhances cytotoxicity of apigenin and liquiritigenin in rat H4IIE hepatoma and C6 glioma cells. Food Chem. Toxicol..

[43] López-Ogalla J., García-Palomero E., Sánchez-Quesada J., Rubio L., Delgado E., García P., Medina M., Castro A., Muñoz P. (2014). Bioactive prenylated phenyl derivatives derived from marine natural products: Novel scaffolds for the design of BACE inhibitors. Med. Chem. Commun..

[44] Fu X., Do T., Schmitz F.J., Andrusevich V., Engel M.H. (1998). New cyclic peptides from the ascidian *Lissoclinum patella*. J. Nat. Prod..

[45] Wang Y.-J., Li Y.-Y., Liu X.-Y., Lu X.-L., Cao X., Jiao B.-H. (2017). Marine antibody-drug conjugates: Design strategies and research progress. Mar. Drugs.

[46] Luesch H., Moore R.E., Paul V.J., Mooberry S.L., Corbett T.H. (2001). Isolation of dolastatin 10 from the marine cyanobacterium Symploca species VP642 and total stereochemistry and biological evaluation of its analogue symplostatin 1. J. Nat. Prod..

[47] Senter P.D., Sievers E.L. (2012). The discovery and development of brentuximab vedotin for use in relapsed hodgkin lymphoma and systemic anaplastic large cell lymphoma. Nat. Biotechnol..

[48] Felczykowska A., Pawlik A., Mazur-Marzec H., Toruńska-Sitarz A., Narajczyk M., Richert M., Węgrzyn G., Herman-Antosiewicz A. (2015). Selective inhibition of cancer cells’ proliferation by compounds included in extracts from Baltic Sea cyanobacteria. Toxicon.

[49] Szubert K., Wiglusz M., Mazur-Marzec H. (2018). Bioactive metabolites produced by *Spirulina subsalsa* from the Baltic Sea. Oceanologia.

