# Supplementary material for: Eighteen New Aeruginosamide Variants Produced by the Baltic Cyanobacterium Limnoraphis CCNP1324
Source: Mar Drugs. 2020 Aug 27;18(9):446. doi: 10.3390/md18090446 (PMC7551963; doi:10.3390/md18090446)
Supplement: Supplementary file 1 [file marinedrugs-18-00446-s001.pdf]

marine drugs

MDPI

Supplementary Material: Eighteen new aeruginosamide variants produced by the Baltic cyanobacterium *Limnoraphis* CCNP1324

Marta Cegłowska[1], Karolia Szubert[2], Ewa Wieczerzak[3], Alicja Kosakowska[1] and Hanna Mazur-Marzec[2*]

[1] Institute of Oceanology, Polish Academy of Sciences, Powstańców Warszawy 55, PL-81712 Sopot, Poland; mceglowska@iopan.pl (M.C.), akosak@iopan.gda.pl (A.K.)
[2] Division of Marine Biotechnology, Faculty of Oceanography and Geography, University of Gdańsk, Marszałka J. Piłsudskiego 46, PL-81378 Gdynia, Poland; karolina.szubert@phdstud.ug.edu.pl
[3] Department of Biomedical Chemistry, Faculty of Chemistry, University of Gdańsk, Wita Stwosza 63, PL-80308 Gdańsk, Poland; ewa.wieczerzak@ug.edu.pl
* Correspondence: hanna.mazur-marzec@ug.edu.pl

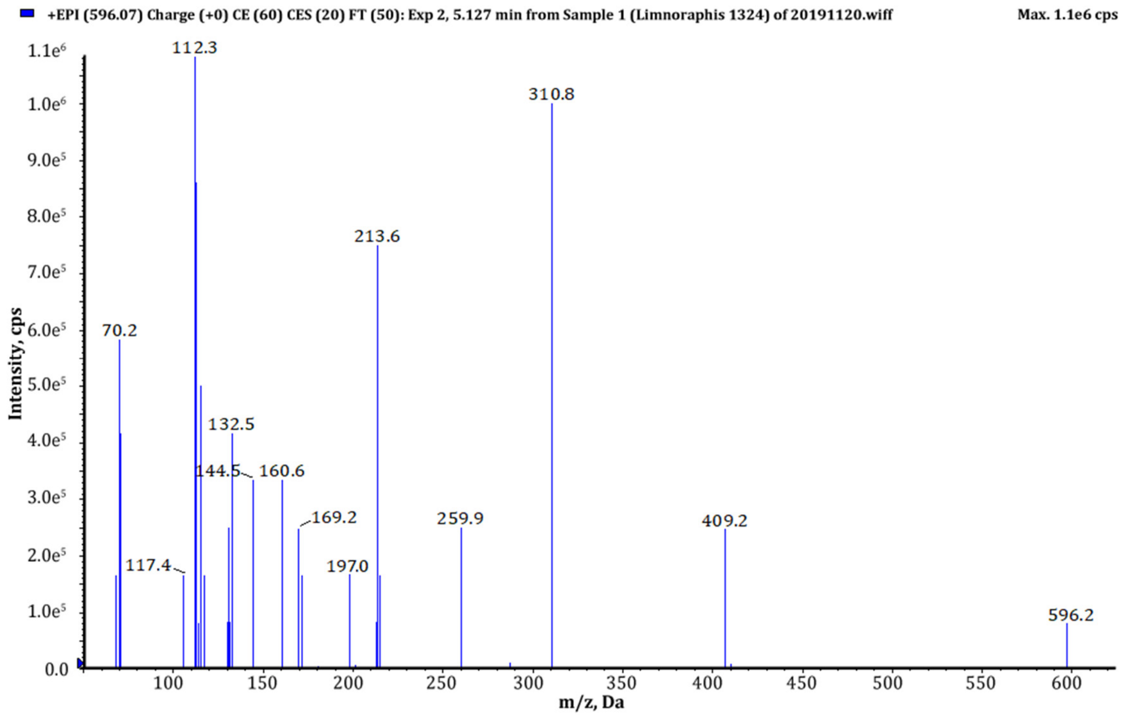

Figure S1. Enhanced product ion mass spectrum of aeruginosamide AER595 with general structure 187+Val+Pro+Pyr+TzlCOOMe identified based on the following fragment ions: 596 [M+H], 409 [Val+Pro+Pyr+TzlCOOMe], 310 [Pro+Pyr+TzlCOOMe+H], 259 [187+Val+H–CO], 213 [Pyr+TzlCOOMe+H], 197 [Val+Pro+H], 169 [Val+Pro+H–CO], 160 [187+H–CO], 144 [TzlCOOMe], 112 TzlCO, 70 Pro immonium ion.

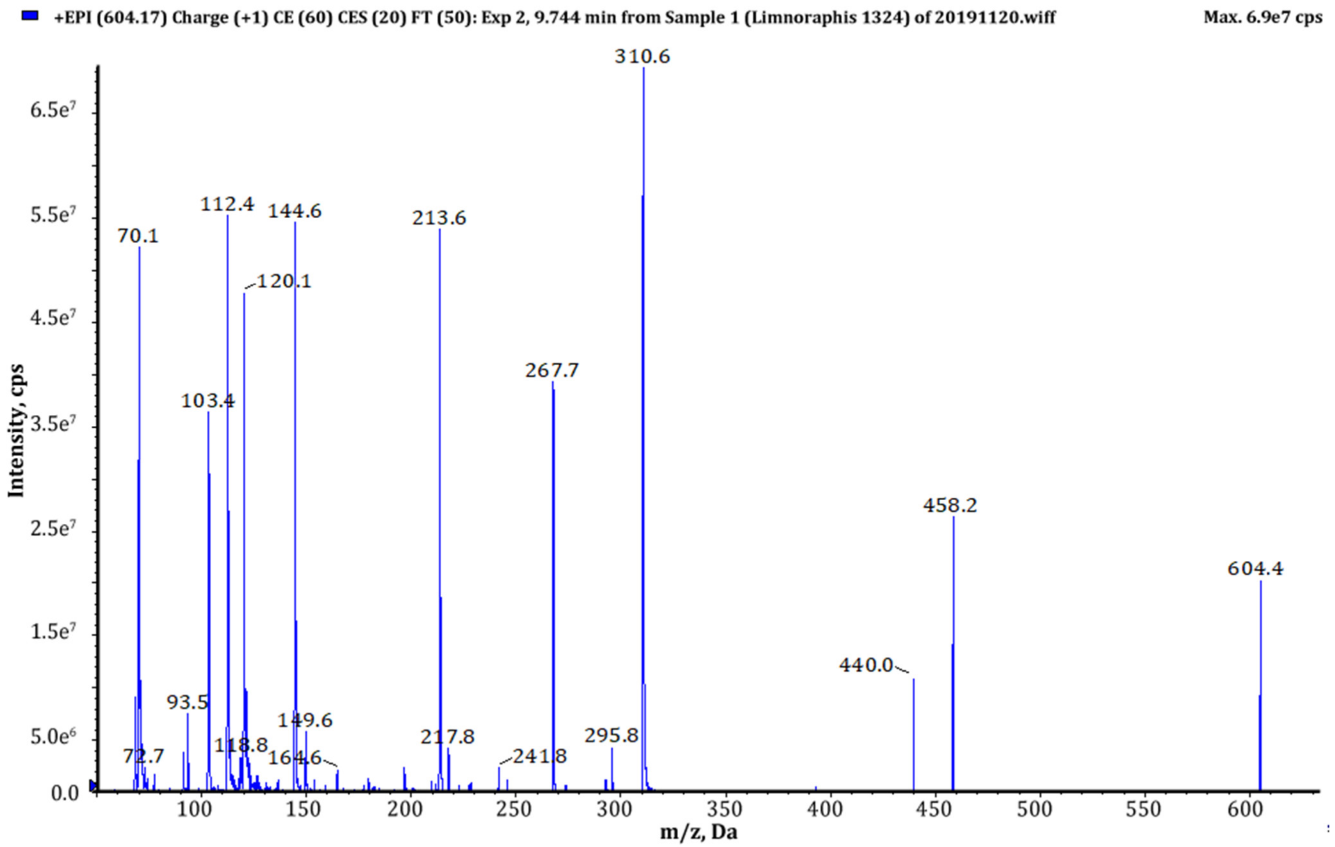

Figure S2. Enhanced product ion mass spectrum of aeruginosamide AEG603 with general structure Phe+Phe+Pro+Pyr+TzlCOOMe identified based on the following fragment ions: 604 [M+H], 458 [M+H–Phe], 310 [Pro+Pyr+TzlCOOMe+H], 295 [Phe+Phe+H], 267 [Phe+Phe+H–CO], 217 [Phe+Pro+H–CO], 213 [Pyr+TzlCOOMe+H], 144 [TzlCOOMe], 120 Phe immonium ion, 112 TzlCO, 70 Pro immonium ion.

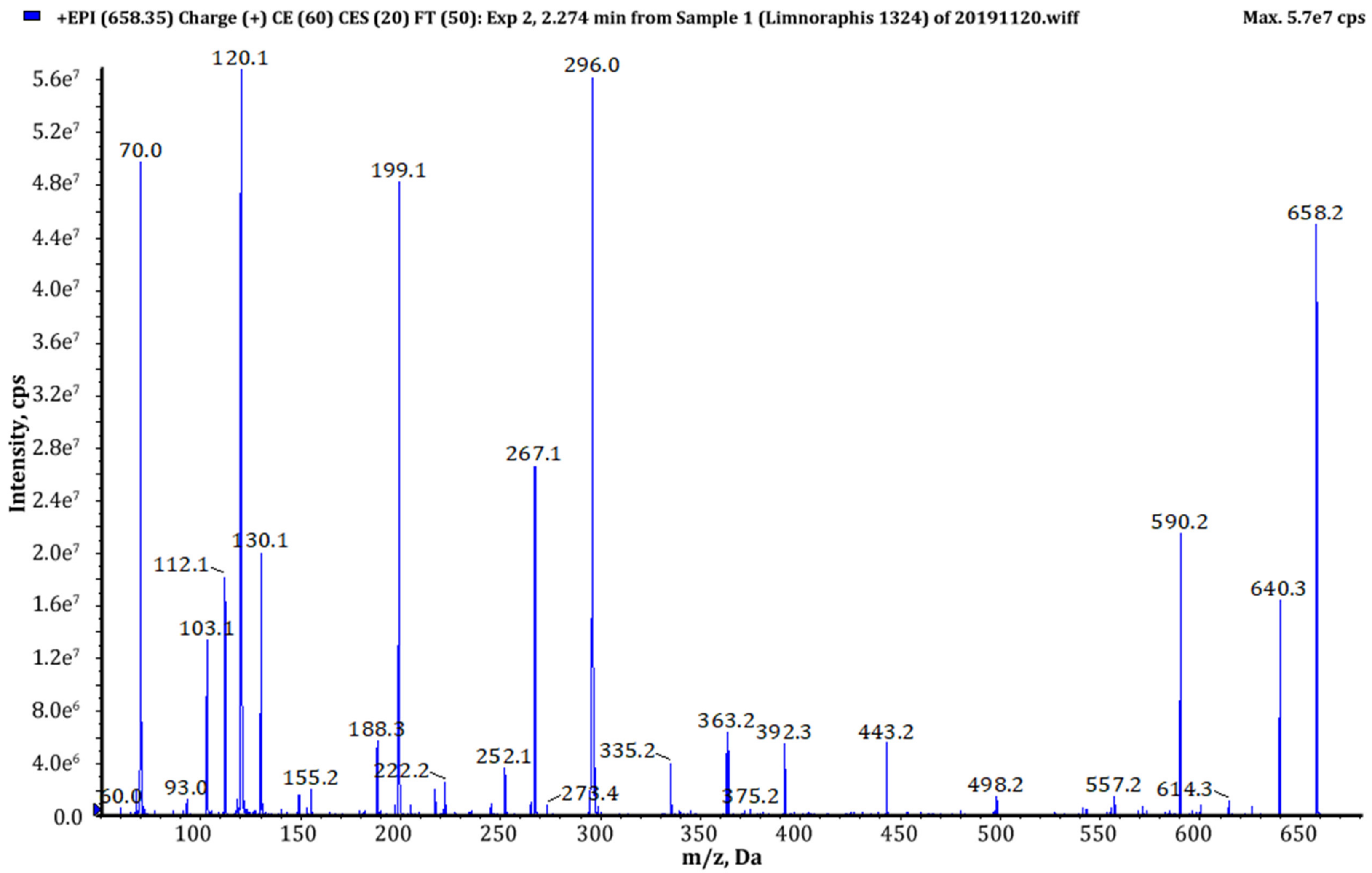

Figure S3. Enhanced product ion mass spectrum of aeruginosamide AEG657 with general structure Pre+Phe+Phe+Pro+Pyr+TzlCOOH identified based on the following fragment ions: 658 [M+H], 640 [M+H–H_2O], 590 [M+H–Pre], 443 [M+H–(Pre+Phe)], 392 [Phe+Phe+Pro+H], 363 [Pre+Phe+Phe+H], 335 [Pre+Phe+Phe+H–CO], 296 [Pro+Pyr+TzlCOOH], 267 [Phe+Phe+H–CO], 199 [Pyr+TzlCOOH+H], 188 [Pre+Phe+H–CO], 130 [TzlCOOH], 120 Phe immonium ion, 112 TzlCO, 70 Pro immonium ion.

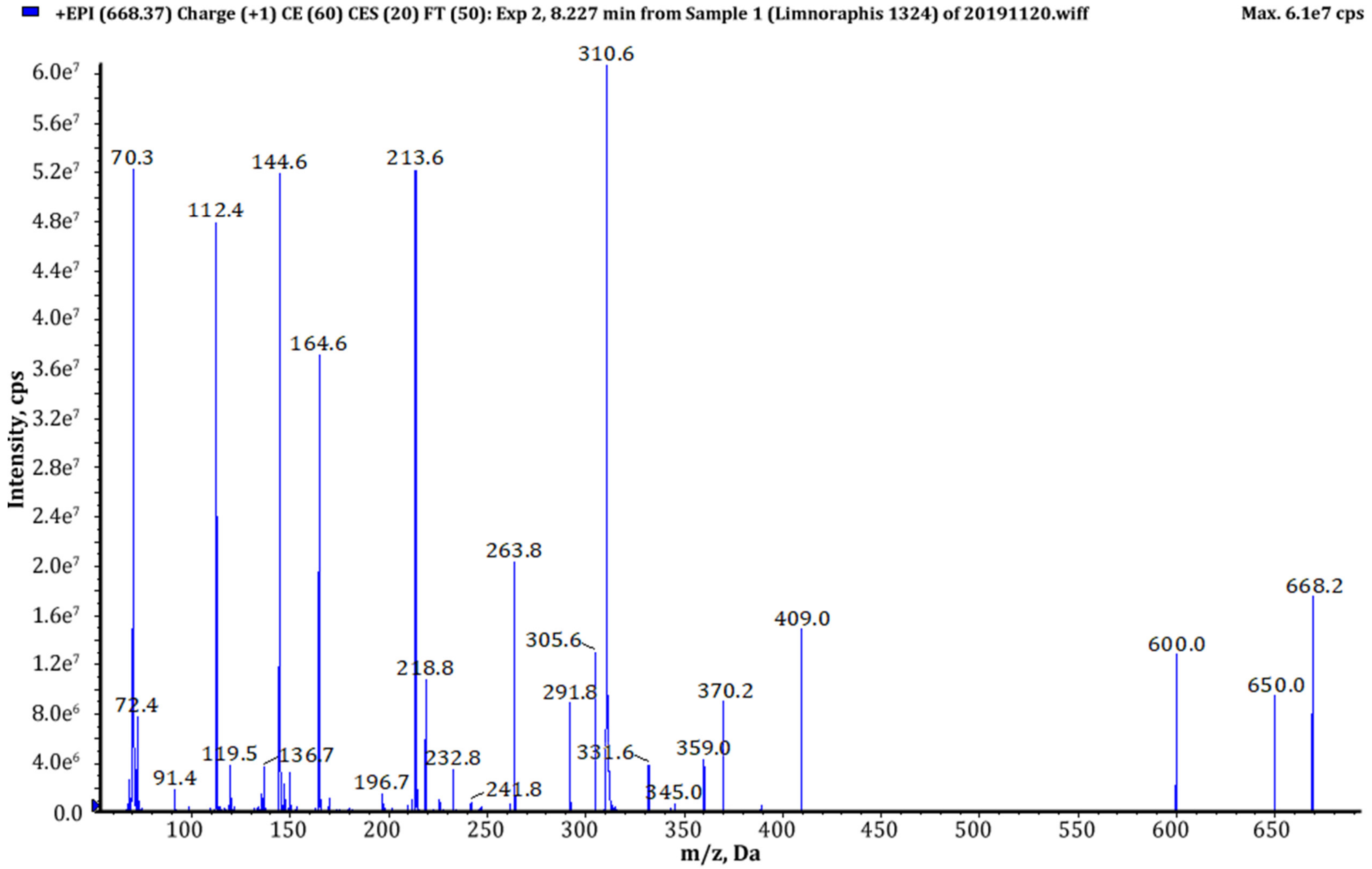

Figure S4. Enhanced product ion mass spectrum of aeruginosamide AER667 with general structure Pre+MeHty+Val+Pro+Pyr+TzlCOOMe identified based on the following fragment ions: 668 [M+H], 650 [M+H–H_2O], 600 [M+H–Pre], 409 [Val+Pro+Pyr+TzlCOOMe], 370 [MeHTyr+Val+Pro+H–H_2O], 359 [Pre+MeHTyr+Val+H], 331 [Pre+MeHTyr+Val+H–CO], 310 [Pro+Pyr+TzlCOOMe+H], 291 [MeHTyr+Val+H], 263 [MeHtyr+Val+H–CO], 213 [Pyr+TzlCOOMe+H], 164 MeHTyr immonium ion, 144 [TzlCOOMe], 112 TzlCO, 72 Val immonium ion, 70 Pro immonium ion.

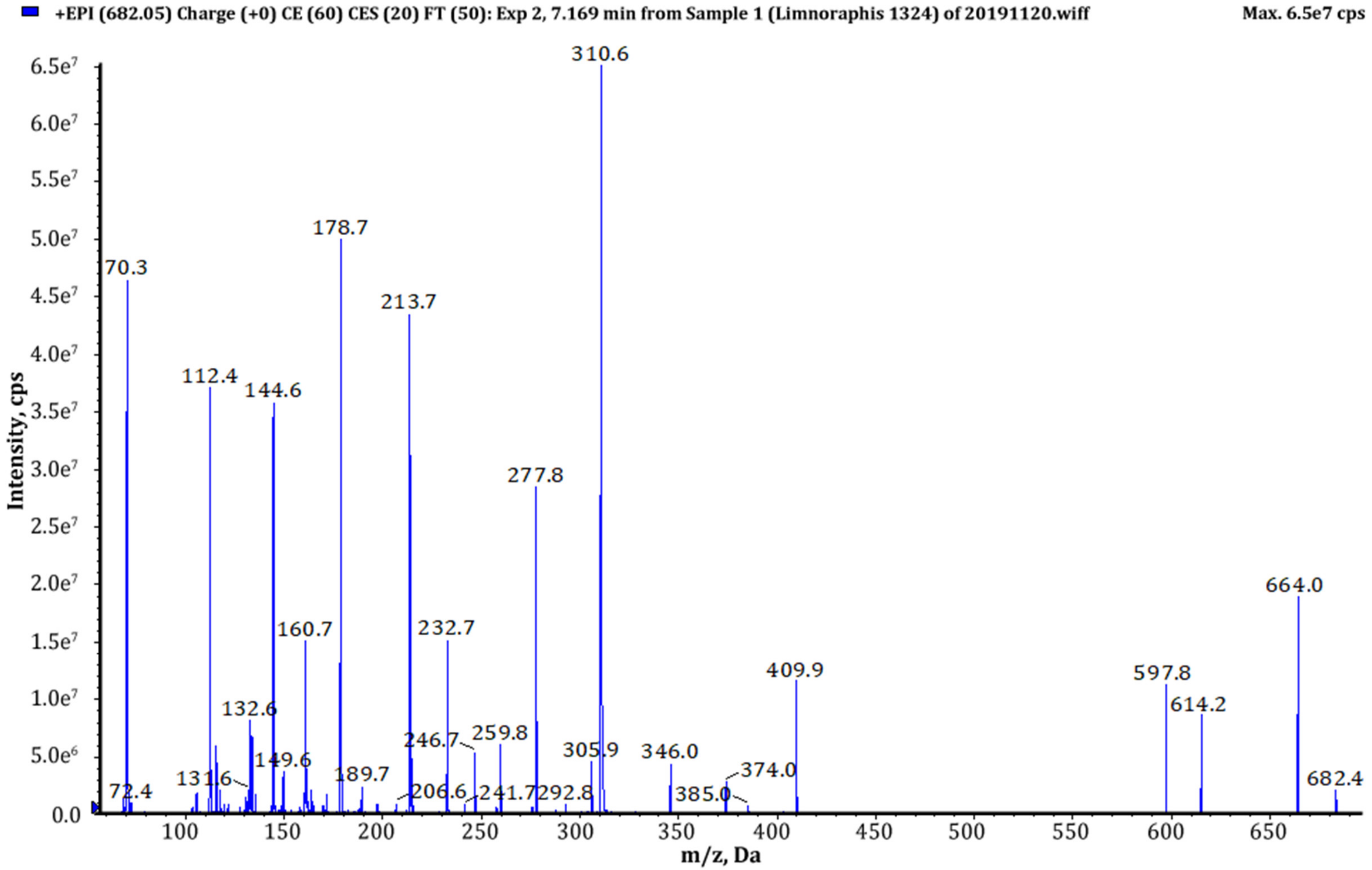

Figure S5. Enhanced product ion mass spectrum of aeruginosamide AER681a with general structure Pre+205+Val+Pro+Pyr+TzlCOOMe identified based on the following fragment ions: 682 [M+H], 664 [M+H–H_2O], 614 [M+H–Pre], 409 [M+H–(Pre+205)], 374 [205+Val+Pro+H–CO], 310 [Pro+Pyr+TzlOMe+H], 305 [205+Val+H], 277 [205+Val+H–CO], 259 [205+Val+H–CO–H_2O], 213 [Pyr+TzlCOOMe+H], 178 [205+H–CO], 144 [TzlCOOMe], 112 TzlCO, 72 Val immonium ion, 70 Pro immonium ion.

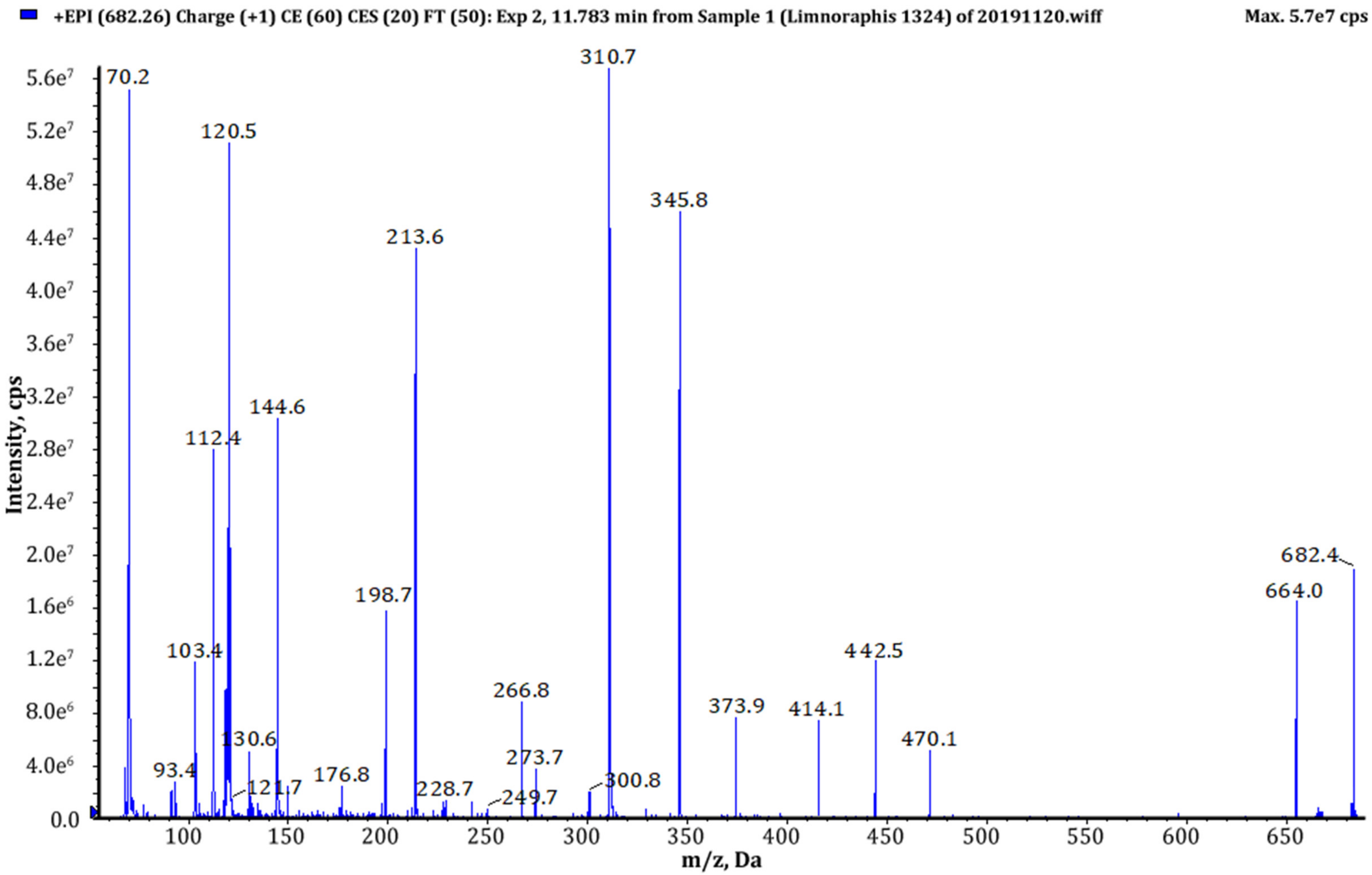

Figure S6. Enhanced product ion mass spectrum of aeruginosamide AER681b with general structure 225+Phe+Pro+Pyr+TzlCOOMe identified based on the following fragment ions: 682 [M+H], 664 [M+H–H_2O], 470 [225+Phe+Pro+H], 442 [225+Phe+Pro+H–CO], 373 [225+Phe+H], 345 [225+Phe+H–CO], 310 [Pro+Pyr+TzlCOOMe+H], 228 [Pre+187+H–CO], 213 [Pyr+TzlOMe+H], 198 [225+H–CO], 144 [TzlCOOMe], 120 Phe immonium ion, 112 TzlCO, 70 Pro immonium ion.

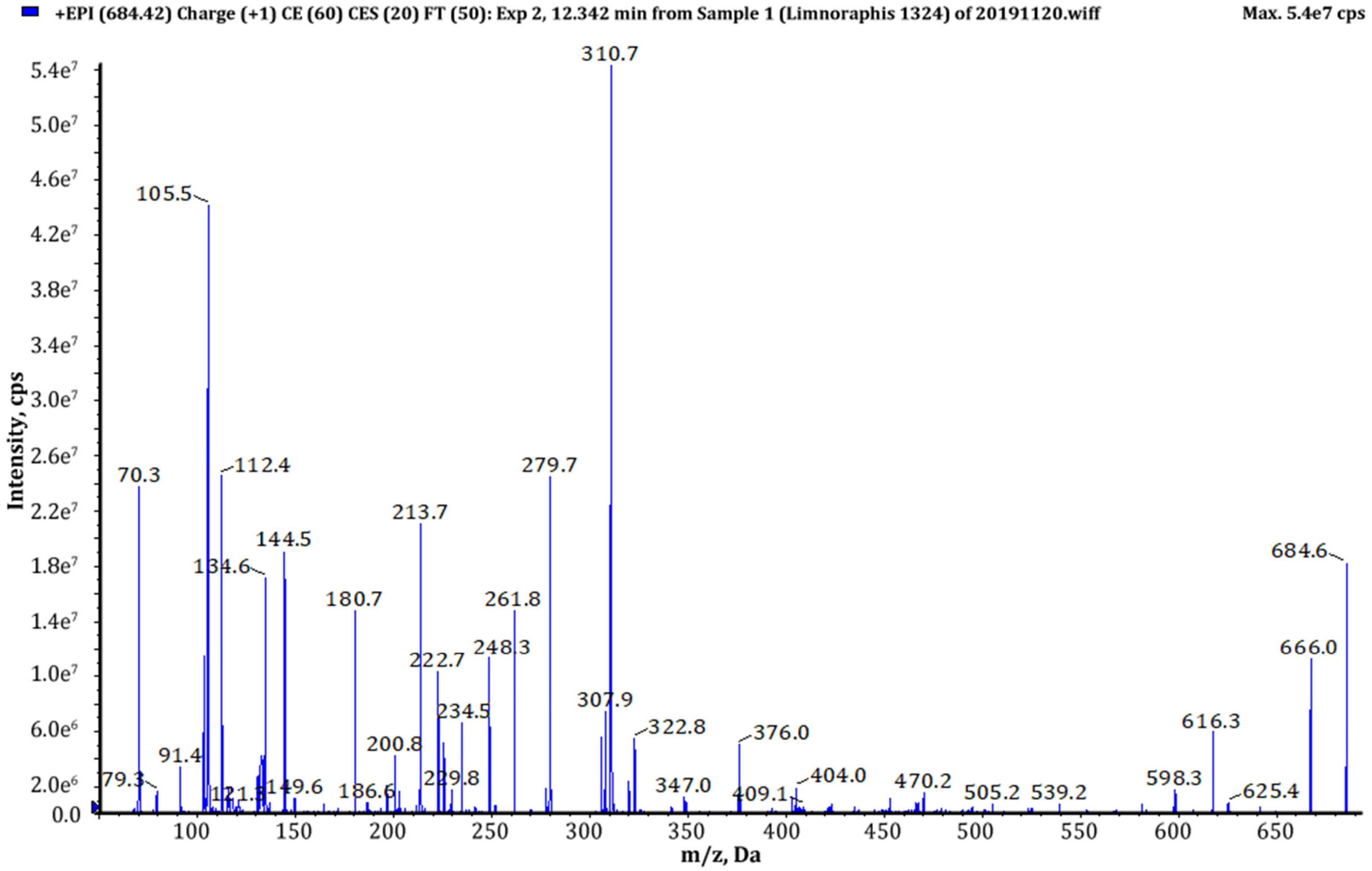

Figure S7. Enhanced product ion mass spectrum of aeruginosamide AER683 with general structure Pre+207+Val+Pro+Pyr+TzlCOOMe identified based on the following fragment ions: 684 [M+H], 666 [M+H–H_2O], 616 [M+H–Pre], 598 [M+H–Pre–H_2O], 409 [Val+Pro+Pyr+TzlCOOMe], 404 [207+Val+Pro+H], 376 [207+Val+Pro+H–CO], 310 [Pro+Pyr+TzlCOOMe+H], 307 [207+Val+H], 279 [207+Val+H–CO], 261 [207+Val+H–CO–H_2O], 213 [Pyr+TzlCOOMe+H], 180 [207+H–CO], 144 [TzlCOOMe], 112 TzlCO, 70 Pro immonium ion.

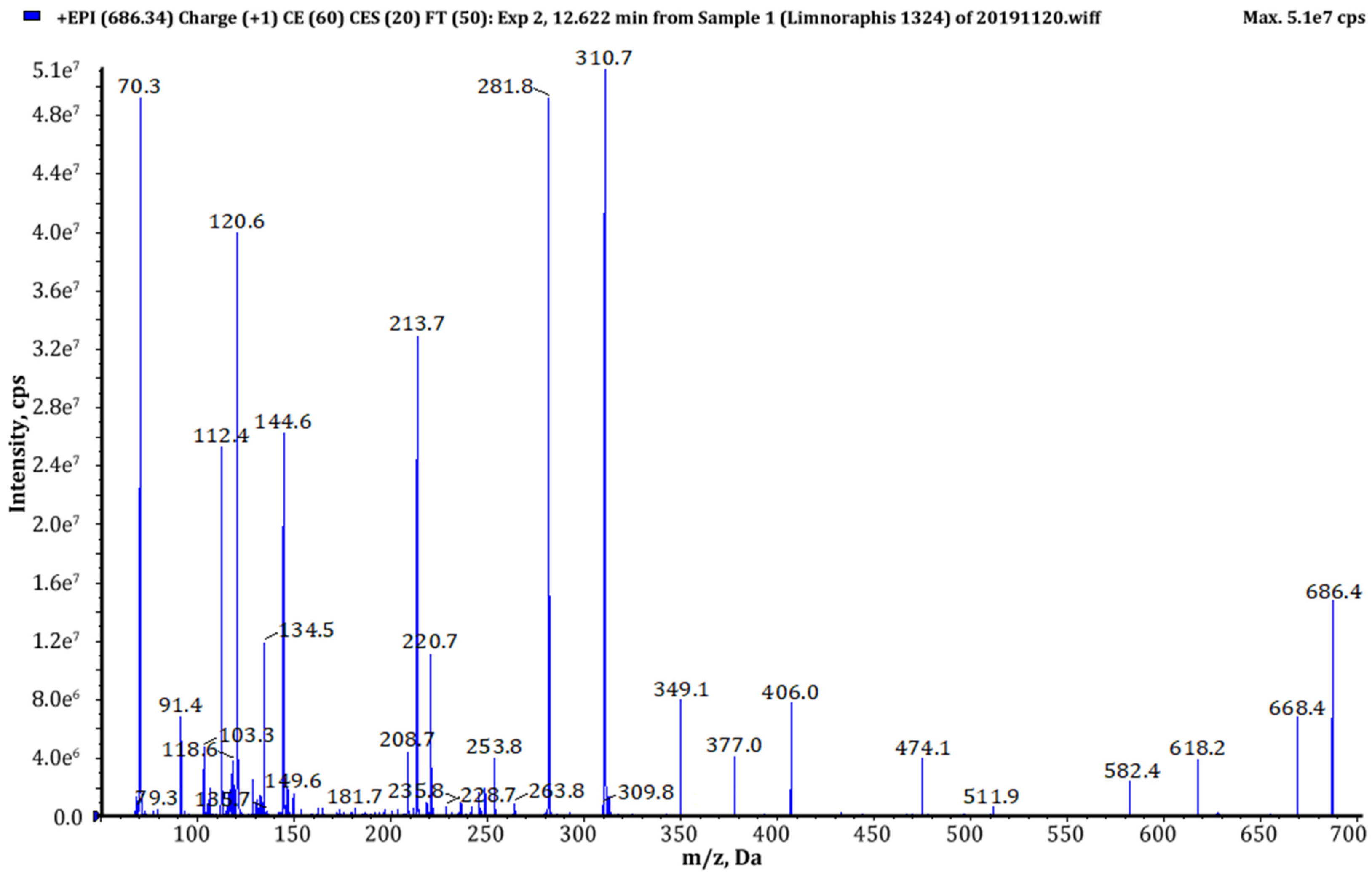

Figure S8. Enhanced product ion mass spectrum of aeruginosamide AER685 with general structure Pre+Phe+Hph/MeHph+Pro+Pyr+TzlCOOMe identified based on the following fragment ions: 686 [M+H], 668 [M+H–H_2O], 618 [M+H–Pre], 474 [Pre+Phe+Hph/MeHph+Pro+H], 406 [Phe+Hph/MeHph+Pro+H], 377 [Pre+Phe+Hph/MeHph+H], 349 [Pre+Phe+Hph/MeHph+H–CO], 310 [Pro+Pyr+TzlCOOMe+H], 309 [Phe+Hph/MeHph+H], 281 [Phe+Hph/MeHph+H–CO], 213 [Pyr+TzlCOOMe+H], 144 [TzlCOOMe], 134 Hph/MeHph immonium ion, 120 Phe immonium ion, 112 TzlCO, 70 Pro immonium ion.

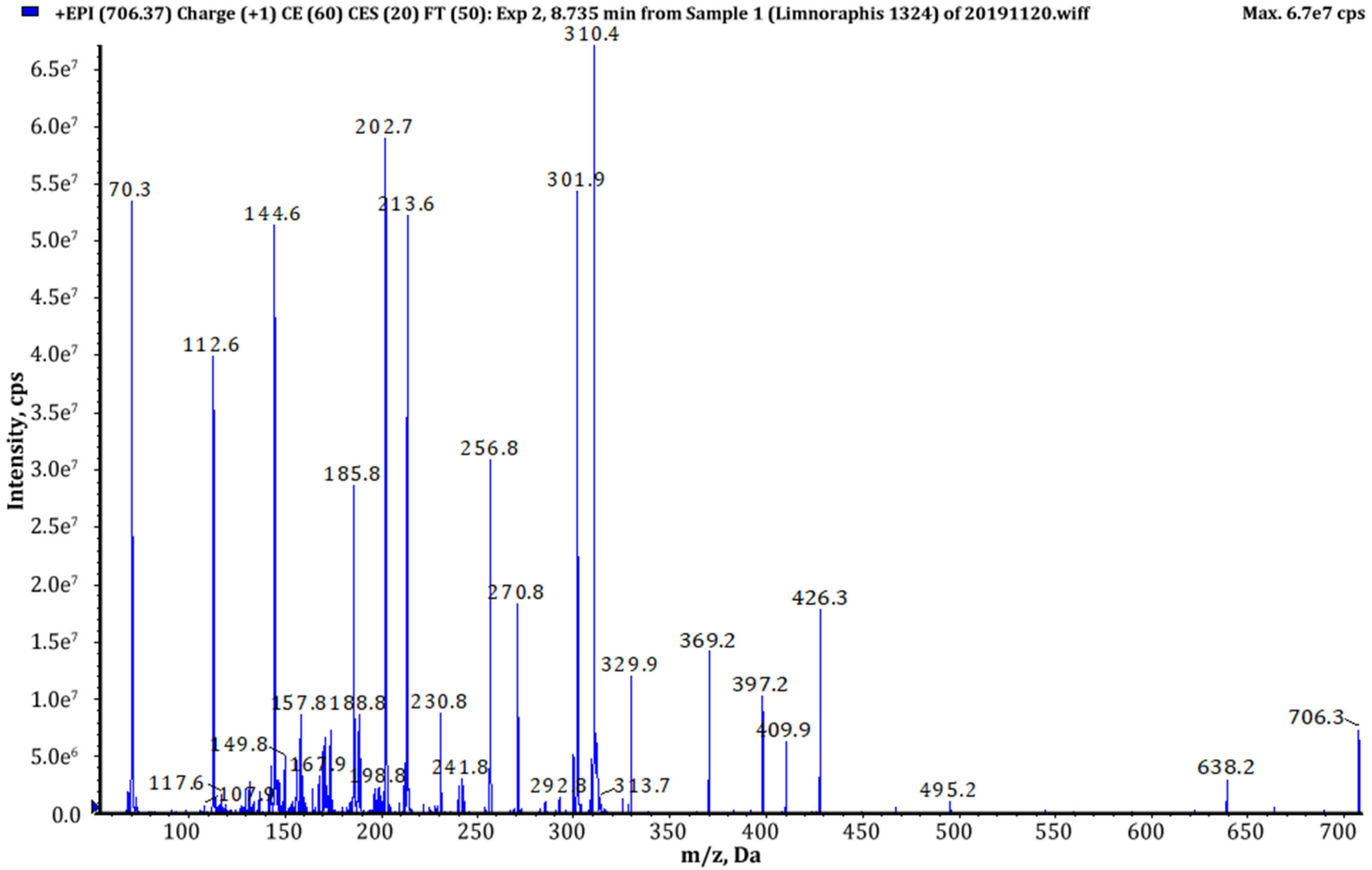

Figure S9. Enhanced product ion mass spectrum of aeruginosamide AEG705 with general structure $(Pre)_2$+Hph/MeHph+Val+Pro+Pyr+TzlCOOMe identified based on the following fragment ions: 706 [M+H], 638 [M+H–Pre], 495 [Pre+Hph/MeHph+Val+Pro+Pyr+H], 426 [Pre+HpH/MeHph+Val+Pro+H], 409 [Val+Pro+Pyr+TzlCOOMe], 397 [$(Pre)_2$+Hph/MeHph+Val+H], 369 [$(Pre)_2$+Hph/MeHph+Val+H–CO], 329 [Pre+Hph/MeHph+Val+H], 310 [Pro+Pyr+TzlCOOMe+H], 301 [Pre+Hph/MeHph+Val+H–CO], 270 [$(Pre)_2$+Hph/MeHph+H–CO], 230 [Pre+Hph/MeHph+H], 213 [Pyr+TzlCOOMe+H], 202 [Pre+Hph/MeHph+H–CO], 144 [TzlCOOMe], 112 TzlCO, 70 Pro immonium ion.

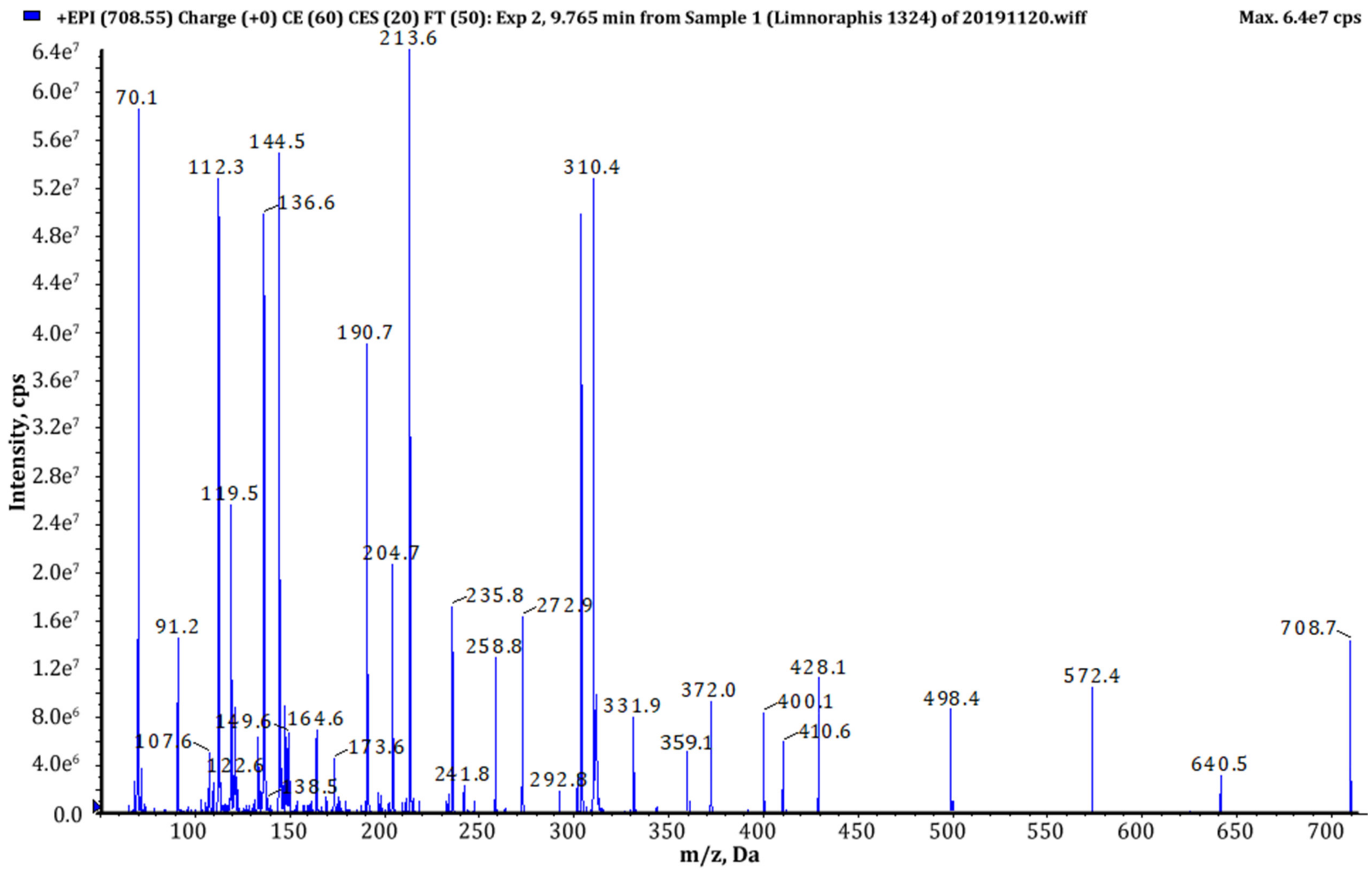

Figure S10. Enhanced product ion mass spectrum of aeruginosamide AEG707 with general structure $(Pre)_2$+Tyr+Val+Pro+Pyr+TzlCOOMe identified based on the following fragment ions: 708 [M+H], 640 [M+H–Pre], 572 [M+H–$(Pre)_2$], 498 [M+H–Pre–TzlCOOMe], 429 [Tyr+Val+Pro+Pyr+H], 409 [Val+Pro+Pyr+TzlCOOMe], 400 [Pre+Tyr+Val+Pro+H–CO], 370 [$(Pre)_2$+Tyr+Val+H–CO], 360 [Tyr+Val+Pro+H], 331 [Pre+Tyr+Val+H], 310 [Pro+Pyr+TzlCOOMe+H], 271 [$(Pre)_2$+Tyr+H–CO], 235 [Tyr+Val+H–CO], 213 [Pyr+TzlCOOMe+H], 204 [Pre+Tyr+H–CO], 144 [TzlCOOMe], 136 Tyr immonium ion, 112 TzlCO, 70 Pro immonium ion.

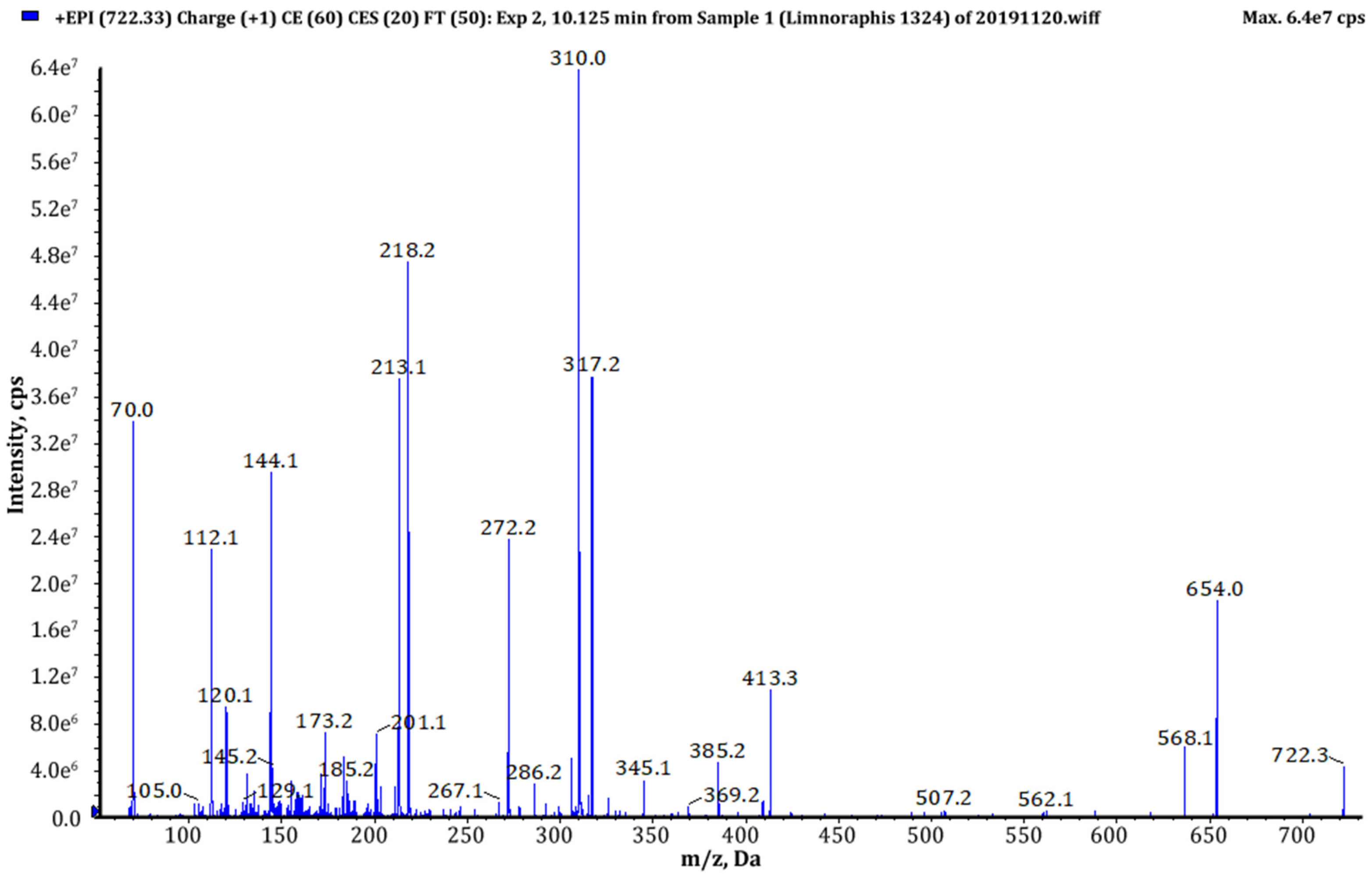

Figure S11. Enhanced product ion mass spectrum of aeruginosamide AEG721 with general structure $(Pre)_2$+Tyr+Ile/Leu+Pro+Pyr+TzlCOOMe identified based on the following fragment ions: 722 [M+H], 654 [M+H–Pre], 586 [M+H–$(Pre)_2$], 413 [$(Pre)_2$+Tyr+Ile/Leu+H], 385 [$(Pre)_2$+Tyr+Ile/Leu+H–CO], 345 [Pre+Tyr+Ile/Leu+H], 317 [Pre+Tyr+Ile/Leu+H–CO], 310 [Pro+Pyr+TzlOMe+H], 272 [$(Pre)_2$+Tyr+H–CO], 213 [Pyr+TzlCOOMe+H], 144 [TzlCOOMe], 112 TzlCO, 70 Pro immonium ion.

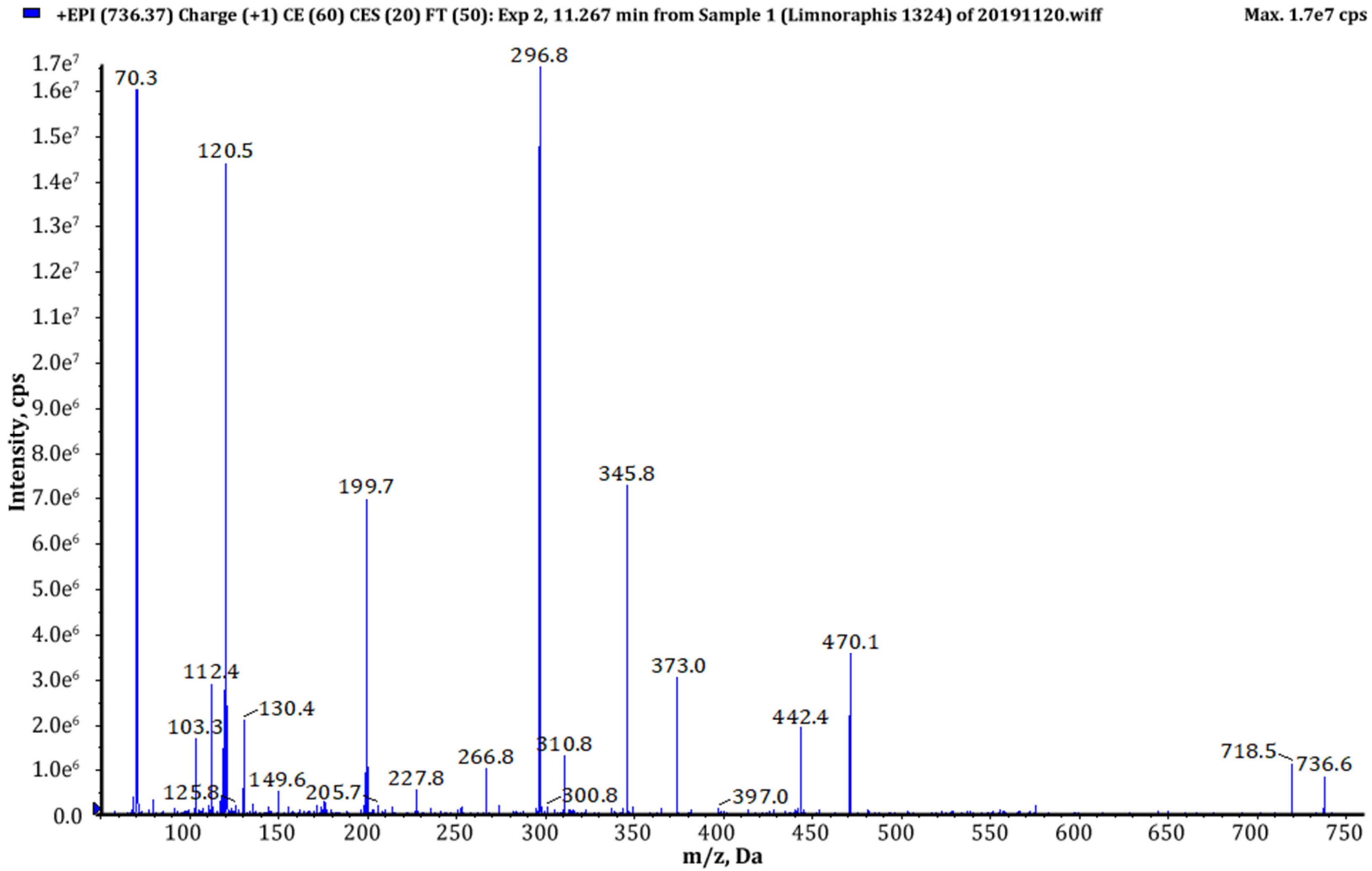

Figure S12. Enhanced product ion mass spectrum of aeruginosamide AER735 with general structure Pre+225+Phe+Pro+Pyr+TzlCOOH identified based on the following fragment ions: 736 [M+H], 718 [M+H–H_2O], 470 [225+Phe+Pro+H], 442 [225+Phe+Pro+H–CO], 373 [225+Phe+H], 345 [225+Phe+H–CO], 296 [Pro+Pyr+TzlCOOH+H], 266 [Pre+225+H–CO], 199 [Pyr+TzlCOOH+H], 130 [TzlCOOH], 120 Phe immonium ion, 112 TzlCO, 70 Pro immonium ion.

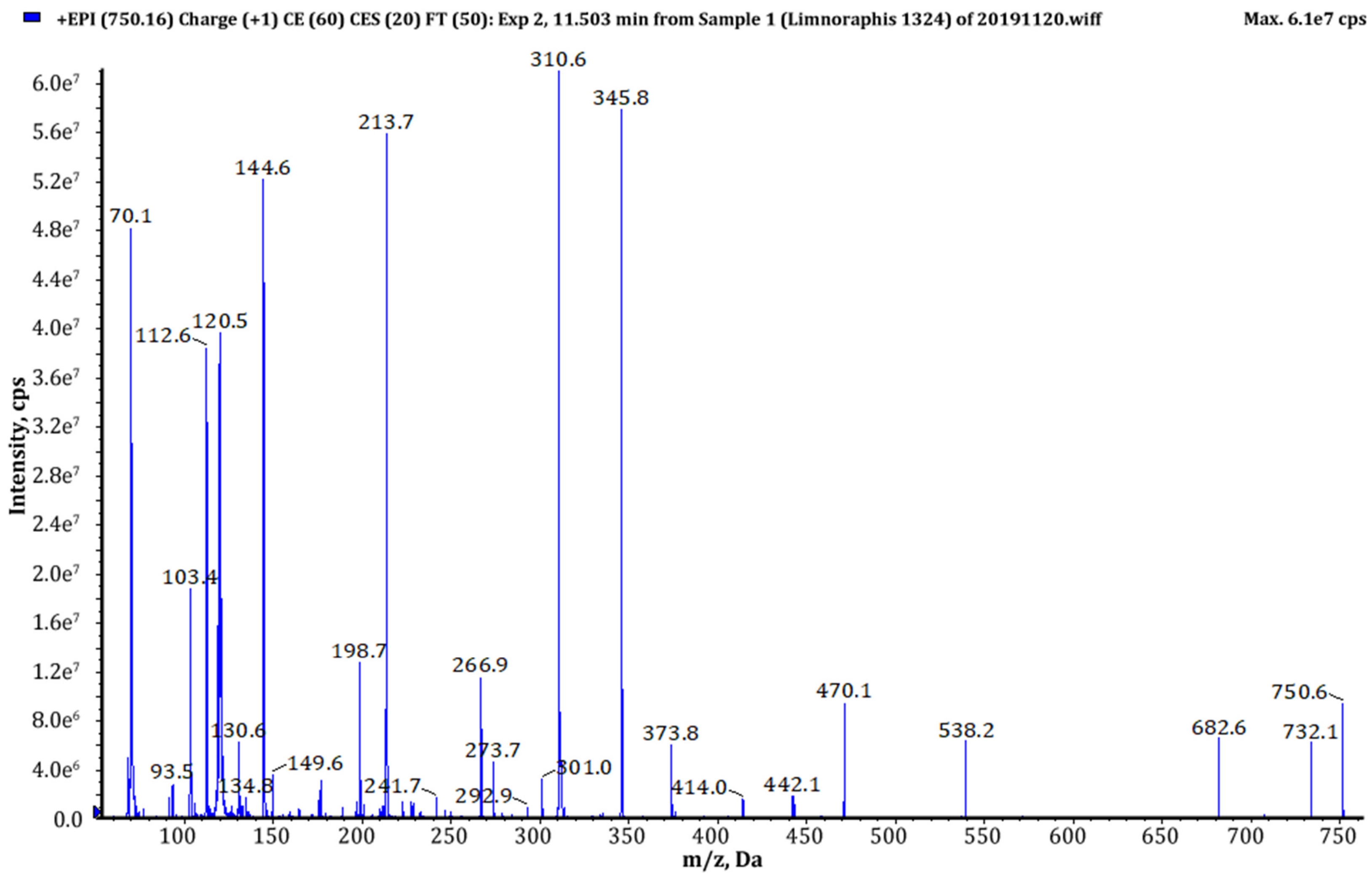

Figure S13. Enhanced product ion mass spectrum of aeruginosamide AER749 with general structure Pre+225+Phe+Pro+Pro+TzlCOOMe identified based on the following fragment ions: 750 [M+H], 732 [M+H–H_2O], 538 [M+H–(Pyr+TzlCOOMe)], 470 [225+Phe+Pro+H], 442 [225+Phe+Pro+H–CO], 373 [225+Phe+H], 345 [225+Phe+H–CO], 310 [Pro+Pyr+TzlCOOMe+H], 266 [Pre+225+H–CO], 213 [Pyr+TzlCOOMe+H], 198 [225+H–CO], 144 [TzlCOOMe], 120 Phe immonium ion, 112 TzlCO, 70 Pro immonium ion.

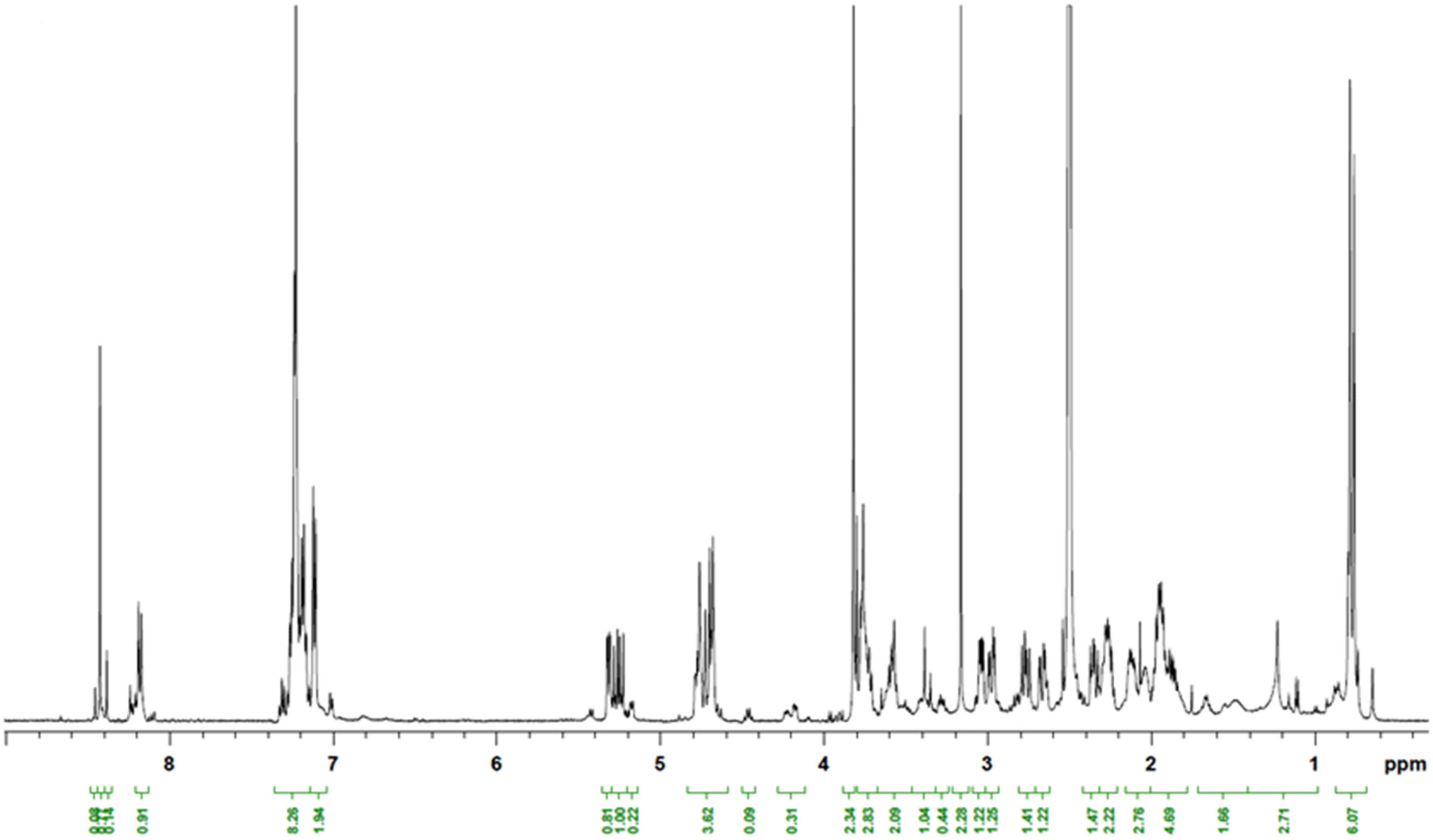

Figure 14. ^{1}H NMR Spectrum of aeruginosamide AEG671 in DMSO-d_6.

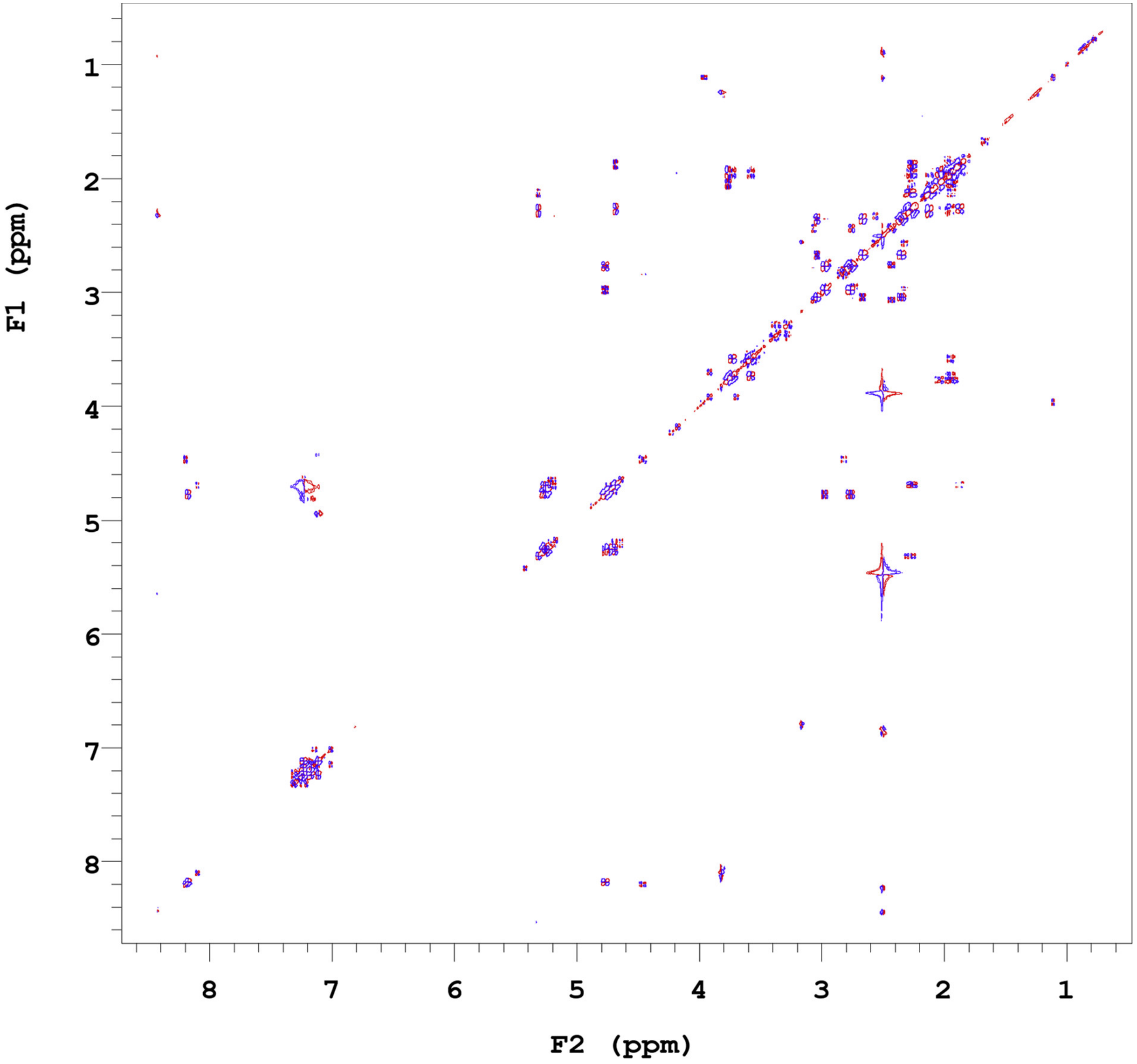

Figure 15. COSY Spectrum of aeruginosamide AEG671 in DMSO-d6.

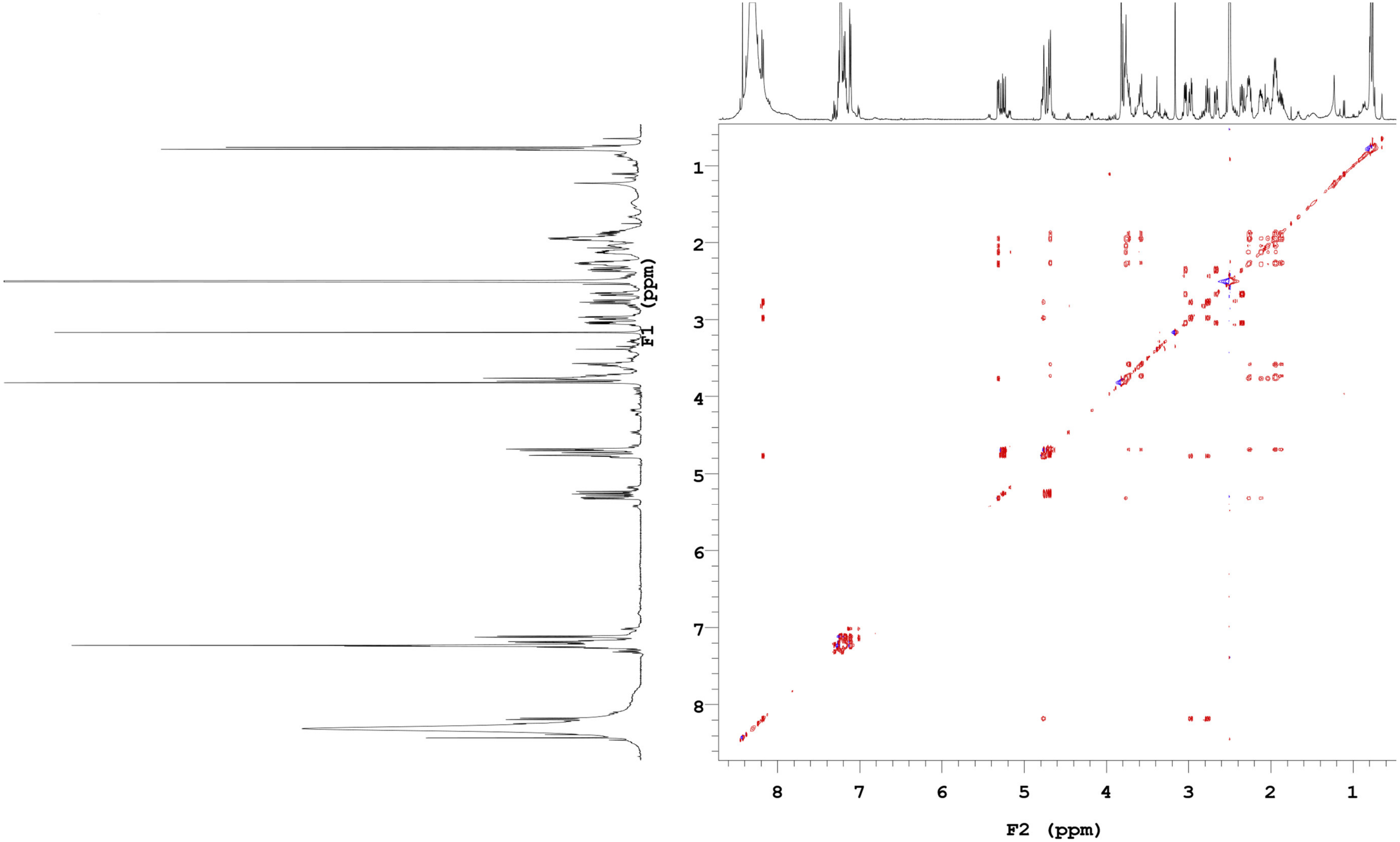

Figure 16. TOCSY Spectrum of aeruginosamide AEG671 in DMSO-d6.

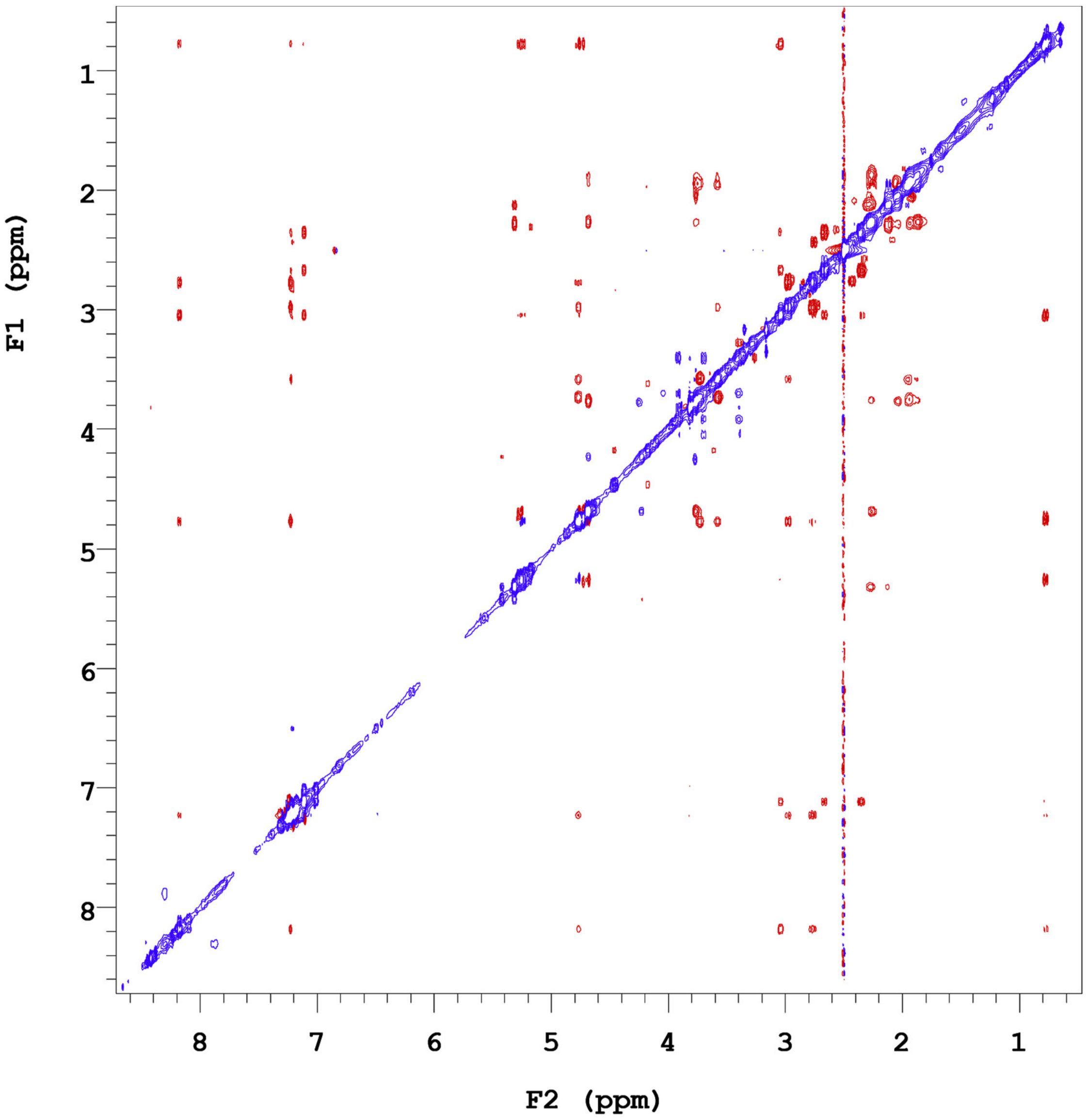

Figure 17. ROESY Spectrum of aeruginosamide AEG671 in DMSO-d_6.

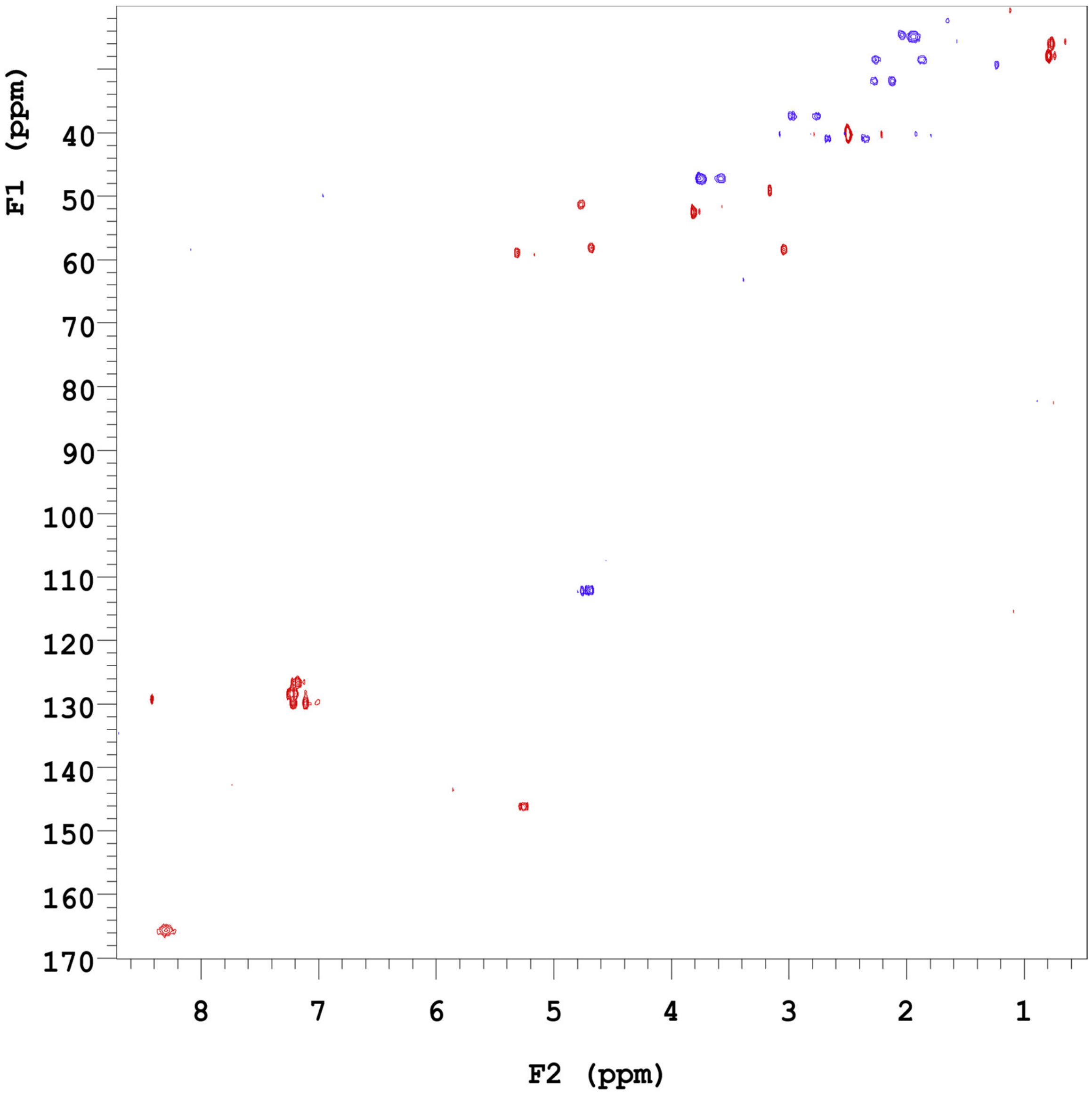

Figure 18. HSQC Spectrum of aeruginosamide AEG671 in DMSO-d_6.

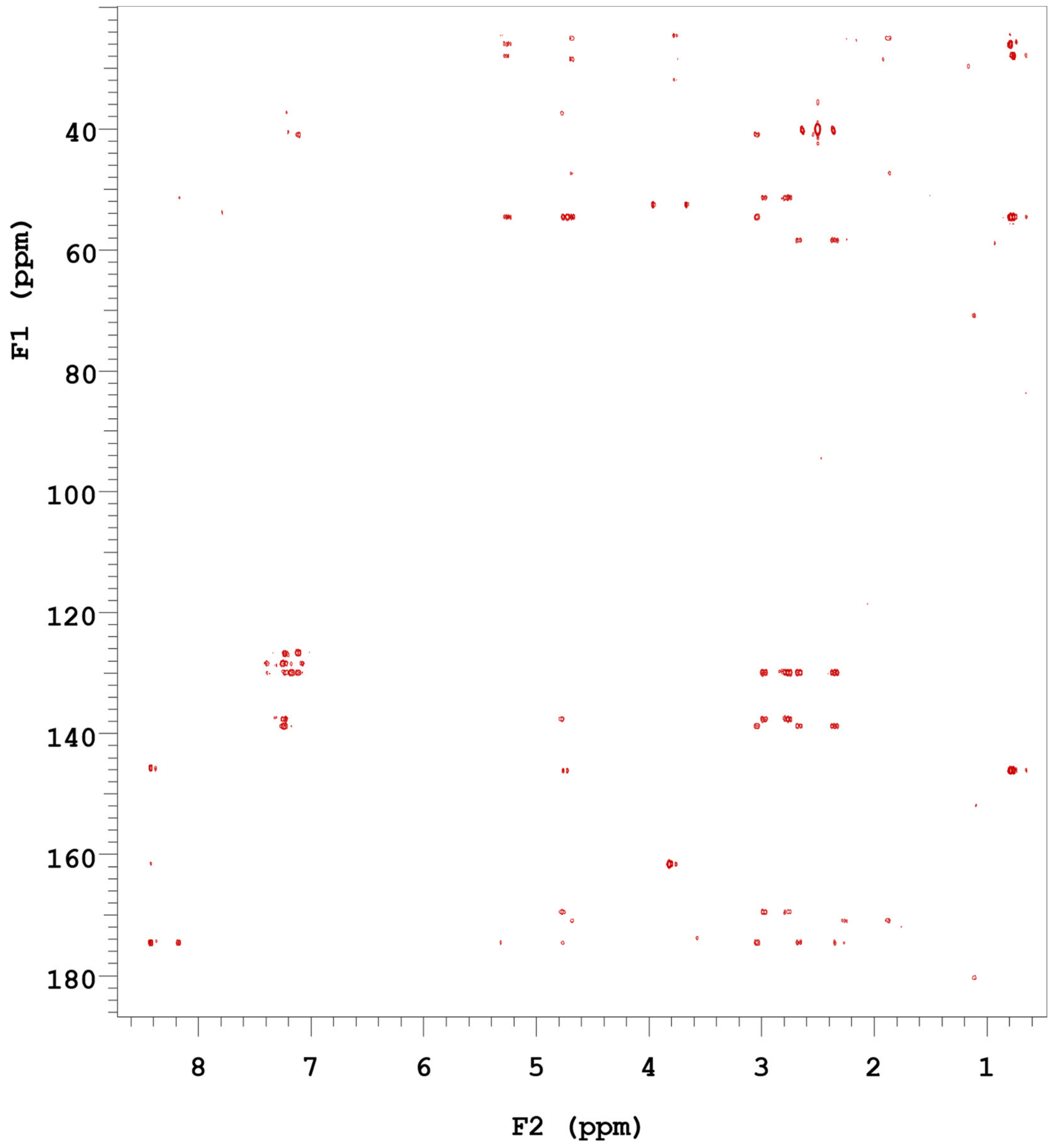

Figure 19. HMBC Spectrum of aeruginosamide AEG671 in DMSO-d_6.

© 2020 by the authors. Licensee MDPI, Basel, Switzerland. This article is an open access article distributed under the terms and conditions of the Creative Commons Attribution (CC BY) license (http://creativecommons.org/licenses/by/4.0/).
